# From beet molasses to malic acid: holistic development of fermentation and downstream process

**DOI:** 10.1186/s13068-026-02736-8

**Published:** 2026-02-03

**Authors:** Luca Antonia Grebe, Christina Maria Krekel, Constantin Alexander Maaß, Mario Beckers, Martin Smotrycki, An N. T. Phan, Lars M. Blank, Katharina Saur, Marcel Mann, Jörn Viell, Andreas Jupke, Jørgen Barsett Magnus

**Affiliations:** 1 AVT.BioVT-Biochemical Engineering, RWTH Aachen University, Forckenbeckstraße 51, 52074 Aachen, Germany; 2 AVT.FVT-Fluid Process Engineering, RWTH Aachen University, Forckenbeckstraße 51, 52074 Aachen, Germany; 3 AVT-NGP2 Biorefinery, RWTH Aachen University, Forckenbeckstraße 51, 52074 Aachen, Germany; 4 Institute of Applied Microbiology-iAMB, Aachener Biology and Biotechnology-ABBt, RWTH Aachen University, Worringer Weg 1, 52074 Aachen, Germany; 5 WSS Research Centre “Catalaix”, Aachen, Germany; 6 IBG-2-Institute for Bio- and Geosciences, Forschungszentrum Jülich GmbH, Johnenstraße, 52428 Jülich, Germany

**Keywords:** *Ustilago trichophora*, Malic acid, Molasses, Bioeconomy, Fed batch, Membrane-based cell retention, Repeated pulsed batch, Downstream processing, Adsorption, Decolorization

## Abstract

**Background:**

The growing demand for sustainable alternatives to fossil-based chemicals has increased interest in platform chemicals derived from renewable biomass sources, such as malic acid. This C4 dicarboxylic acid is valued for its diverse application potential in food, pharmaceuticals, and bioplastics. Sustainable platform chemicals remain commercially uncompetitive primarily due to high production costs driven by high substrate costs. Microbial production using more cost-effective feedstocks like sugar beet molasses shows promise. However, it faces challenges from high osmolality, growth inhibitors, and predetermined substrate composition during fermentation, as well as elevated pigmentation that complicates downstream processing. Moreover, the separation techniques typically used for highly polar carboxylic acids face considerable yield limitations due to the high solubility of malic acid and its salts.

**Results:**

This study developed an all-encompassing production process for malic acid from untreated sugar beet molasses. Fermentative malic acid production with *Ustilago trichophora* was investigated in batch, fed-batch, and pulsed batch in shake flask scale, followed by a scale-up into 150 L pilot scale. A total of 15.7 kg malic acid was produced in a repeated pulsed batch with membrane-based cell retention with a titer of 108 g/L, a yield of 0.50 g/g, and a space–time yield of 0.66 g/L/h (max. 1.1 g/L/h). In addition, the byproduct succinic acid was detected in concentrations of up to 22.9 g/L. In the subsequent downstream processing, activated carbons were used for two-stage product capture, solvent change, and decolorization, followed by crystallization of the products malic acid and succinic acid. Based on experimental results, an Aspen Plus model was developed to estimate the overall process yields of 0.43 g malic acid (98% purity) and 0.10 g succinic acid per gram sucrose equivalent. A techno-economic analysis suggests production costs within the range of current market prices.

**Conclusion:**

Agricultural residue streams are often proposed as cost-effective alternatives for fermentative platform chemical production, although the challenges addressed hamper the direct transfer of process strategies from established organic acid production. By presenting a holistic approach explicitly tailored to malic acid production from untreated molasses, this work demonstrates the techno-economic feasibility of the developed process at a meaningful scale.

**Supplementary Information:**

The online version contains supplementary material available at 10.1186/s13068-026-02736-8.

## Background

The increasing demand for sustainable alternatives to fossil-based chemicals has driven significant interest in platform chemicals derived from renewable biomass sources, such as carboxylic acids, amines, and alcohols. These versatile compounds can be converted into a variety of high-value chemicals and materials [1]. Malic acid, a C4 dicarboxylic acid with two pK_a_ values at pH 3.40 and pH 5.11 and a hydroxyl group at the second carbon atom, is a prominent candidate due to its diverse functional properties and was previously listed as one of the twelve most promising biobased building block chemicals by the US Department of Energy [2, 3]. It is currently produced petrochemically by hydration of maleic anhydride, resulting in a racemic mixture of D- and L-malic acid [4]. In 2024, the market comprised a volume of US$239 million, with an expected growth of 5.2% per year until 2030 [5]. Malic acid is mainly used as an acidulant and flavor enhancer in the food and beverage sector, but also holds potential as a key building block for the synthesis of both commodity and specialty chemicals, as well as for the application in the pharmaceutical and cosmetic sector [2, 4, 6, 7]. The 1,4-dicarboxylic acid can be converted into biodegradable polyesters and polyamides and could serve as a replacement for petroleum-derived maleic acid, which is widely used as a monomer in unsaturated polyester resins and as a precursor to a variety of molecules, such as 1,4-butanediol [8]. Biotechnological malic acid production yields the enantiopure L-isomer, preferred for polymerization [4], making it not only more sustainable but also potentially advantageous compared to petroleum-derived racemic malic acid.

### Malic acid fermentation on renewables

Biotechnological malic acid production on renewables has already been studied in the literature, although high costs compared to the petrochemical production route remain challenging [4]. One of the major cost drivers in fermentative production is the substrate costs [9]. A transition from pure sugars to fermentable residue streams can lower substrate costs and reduce competition with food resources. Product titers obtained on complex residue streams are typically lower than on pure sugars, and processes can be categorized based on required pretreatment. For the highest titers, yield and space–time yield (STY) are given in parentheses. Lignocellulose-based biomass usually requires a two-step process comprising biomass hydrolysis and subsequent fermentation, with titers of 38 g/L on beech wood cellulose hydrolysate [10], 32 g/L on corncob hydrolysate [11], or 77 g/L corn hull hydrolysate [12]. One-step processes have also been conducted and reached titers of 17 g/L on thin stillage [13] or 105 g/L on milled corncob (0.4 g/g total plant biomass, ~ 0.66 g/L/h) [14].

Molasses, a byproduct from sugar production, is another promising cost-effective substrate. It has a high availability in the Rhenish region of Germany, and with its high sucrose content of about 650 g/L, it is currently mainly used as animal feed [4, 15]. With a price of about 260 EUR/t_sucrose equivalents_, molasses is more than twice as cheap as sugar, based on personal communication with suppliers in the German market in 2025. Although the sugars in molasses are readily available for sucrose-utilizing strains, this residue stream introduces challenges such as high osmolality and the presence of metal ions or organic acids (e.g., lactic acid) that may inhibit microbial growth [15–17]. For sugarcane molasses, the highest titer of 111 g/L (0.32 g/g sucrose, ~ 0.93 g/L/h) was only reached after phosphoric acid treatment, which reportedly reduced inhibitory iron and copper levels [18]. In another study, sugarcane molasses was pretreated in three steps with tricalcium phosphate, potassium ferrocyanide, and sulfuric acid, yielding 95.4 g/L polymalic acid (corresponding to 109.07 g/L malic acid, 0.28 g/g, 0.65 g/L/h) [19]. Molasses derived from sugar beets naturally contains lower levels of these heavy metals [15, 20], and can potentially be used without pretreatment, which would be beneficial for the overall process costs.

The Ustilaginaceae, a family of smut fungi, are well known for their broad substrate range and resilience to impurities [21, 22]. In combination with their naturally broad product spectrum and unicellular, yeast-like growth, they are ideal candidates for sustainable platform chemical production [23]. For malic acid production, the wild-type strain *Ustilago trichophora* has already been identified as a promising production host [24], showing both growth and production on molasses at neutral pH values [25]. *U. trichophora* likely produces malic acid via the oxidative citric acid cycle (TCA), which has a theoretical maximal yield of 0.78 g/g (2 mol/mol) malic acid per sucrose [21, 24]. The product is subsequently secreted by the organism, and the deprotonated organic acid accumulates in the fermentation broth [21]. The resulting product inhibition is a common limitation in the microbial production of organic acids. For *U. trichophora*, product inhibition above 100 g/L malic acid was previously reported [21].

### Downstream processing strategies

Several strategies for the downstream processing of malic acid are discussed in the literature. A central challenge is its high solubility in water. One approach to purifying malic acid is to precipitate it as calcium malate from the neutral fermentation broth after cell removal [26]. This strategy is established on an industrial scale for the biotechnological production of citric acid, which, like malic acid, is highly water soluble. However, the high solubility of calcium malate (12.2 g/L) compared to calcium citrate (0.96 g/L), significantly lowers precipitation yields [4, 27]. Beyond precipitation, direct crystallization from fermentation broth has been used as a robust separation step at an industrial scale for other dicarboxylic acids, such as itaconic acid [28]. However, as malic acid is highly soluble in water, direct crystallization, as used, e.g., for itaconic acid production [29, 30], would entail high energy demands for water evaporation [31]. Moreover, the high solubility of malic acid leaves substantial residual acid in the mother liquor, reducing overall yield, while the accumulation of co-salts limits further concentration [30]. Alternative purification strategies include reactive extraction with trioctylamine [32–35] and ion exchange adsorption [36–38]. Although reactive extraction is a promising strategy for purifying highly soluble acids, using organic solvents such as octanol in biorefinery processes presents challenges in terms of processing, disposal, and safety [39]. In addition, extractants such as trioctylamine are known to co-extract inorganic salts [39]. The high osmolarity of the fermentation broth thus impairs the extraction performance. Similar limitations apply to ion exchange, despite the high capacity of ion exchangers for malic acid. [38, 40].

In the recent years, the adsorption of carboxylic acids onto non-polar adsorbents, such as activated carbons, has gained increasing attention [41–44]. At low pH, activated carbons exhibit high capacities for carboxylic acids [41]. They interact with both the carbon skeleton of the activated carbons, as well as functional groups formed by heteroatoms integrated into the carbon skeleton's surface [45, 46]. Thereby, the degree of functionalization depends on the activation method and the precursor material used [46]. As pH increases, capacity decreases because deprotonation to carboxylate anions reduces affinity for the nonpolar surface [45]. Activated carbons have also proven to be a robust separation option for purifying dicarboxylic acids from fermentation broths, for example, in in situ product removal applications [43]. However, in the present case, in situ removal is not feasible due to mismatched pH requirements between fermentation and downstream processing, as well as the high osmolarity of the broth. After adsorption, the carboxylic acid is typically eluted with a solvent such as ethanol, thereby enabling a solvent switch to a volatile solvent in which malic acid is less soluble [43].

### Conceptual process design

**Fig. 1:**
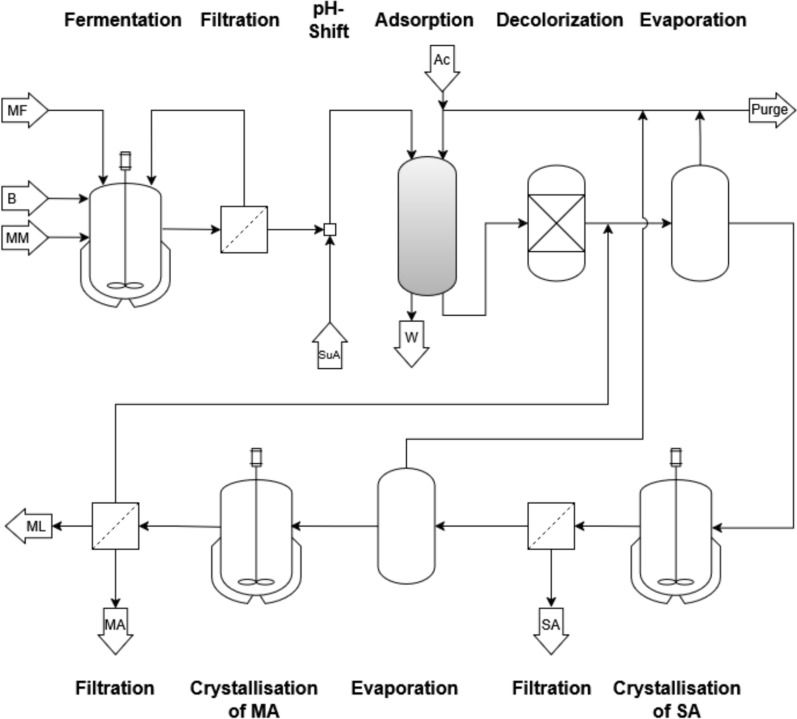
Proposed process flow sheet for malic acid production from molasses. Molasses serves as the substrate in the initial medium (MM) and as the feed (MF). During fermentation, the pH is maintained at 6.5 by the addition of base (B). After fermentation, cells are removed from the broth by filtration. The filtrate is acidified to pH 2 with sulphuric acid (SuA), and malic and succinic acid are captured on an activated‑carbon column by adsorption. The residual broth is discharged as waste (W). The adsorbed carboxylic acids are eluted with acetone (Ac) and subsequently decolorized. The eluate is concentrated by evaporating acetone until the solubility limit of malic acid is reached. Succinic acid (SA) is crystallized and removed from the mother liquor. The mother liquor is further concentrated, leading to crystallization of malic acid (MA), which is separated from the mother liquor (ML) by filtration. The evaporated acetone and the mother liquor are recycled, with small purge streams applied

The overall target of this work is the biotechnological production of pure, crystalline malic acid from untreated sugar beet molasses. In previous works, small-scale batch cultivation with molasses as the sole carbon source has successfully been established for the wild-type strain *U. trichophora* [25]. In addition to malic acid, this strain produces succinic acid as a by-product. Building on the established cultivation protocol [25], the fermentation process is optimized regarding the key performance indicators (KPIs) titer, yield, and STY. As high initial molasses concentrations might inhibit growth, substrate feeding is implemented to increase malic acid titers. Two feeding modes are evaluated, and the technical feasibility is demonstrated at 150 L pilot scale. Following fermentation, the cellular biomass is removed from the broth by filtration. The pH is then shifted from pH 6.5 to pH 2.0 to protonate malic acid prior to adsorption onto activated carbon. This adsorption step serves as the first purification step, removing the high salt load and enabling a solvent change from water to a short-chain solvent. The chosen solvent should preferably have a lower boiling point and lower solubility for malic acid than water. Previous studies have used ethanol for this purpose [43]. However, as malic acid reacts with alcohols to form esters, acetone is employed as the eluent in this study. Acetone has an even lower boiling point and does not form an azeotrope with water, thereby improving solvent recovery. The resulting eluate contains malic and succinic acid dissolved in acetone. This is subsequently decolorized using a different type of activated carbon. To separate malic acid from the succinic acid by-product, a two-stage crystallization process is applied. Given that succinic acid is less soluble than malic acid in acetone, succinic acid is crystallized first. Before the first crystallization, the eluate is concentrated at elevated temperatures until the solubility limit of malic acid at the crystallization temperature of 10 °C is almost reached. Subsequent cooling to 10 °C leads to the selective crystallization of succinic acid. The solids are then removed by filtration, while the mother liquor is concentrated even further, surpassing the solubility limit of malic acid. After crystallization of malic acid, the solid product is recovered via filtration. Both the mother liquor and the eluent acetone are recycled. Figure 1 depicts the proposed production process of malic acid from sugar beet molasses.

This work demonstrates the proposed process concept and optimizes each unit operation. All steps up to and including decolorization were investigated experimentally using fermentation broth, whereas crystallization was examined only in pure-component model solutions. Based on these experimental data, a process simulation was conducted to determine the overall process yield and final product purity. The results then served as the basis for estimating operating costs.

## Materials and methods

### Microorganism

All experiments were performed with the strain *Ustilago trichophora* TZ1, which will henceforth be abbreviated to *U. trichophora* [24]. The strain was stored as a cryo culture containing 0.7 mL fermentation broth and 0.3 mL glycerol solution (500 g/L) at −80 °C.

### Cultivation media

The preculture medium was prepared according to Pastoors et al*.* [43]. All chemicals were sourced from either Carl Roth GmbH + Co. KG (Karlsruhe, Germany), Merck KGaA (Darmstadt, Germany), or VWR International (Radnor, PA, United States). Beet molasses from Pfeifer & Langen GmbH & Co. KG (Jülich, Germany) was used, which was previously analyzed by Helm et al. [15]. The preculture medium was prepared without molasses and consisted of 50 g/L sucrose, 4 g/L NH_4_Cl, 2 g/L KH_2_PO_4_, 0.4 g/L MgSO_4_ • 7 H_2_O, 0.01 g/L FeSO_4_ • 7 H_2_O, 1 mL/L trace element solution, and 0.1 M 2-(N-morpholino)ethanesulfonic acid (MES) buffer (pH 7.2), if not specified otherwise. The trace element solution contained 15 g/L ethylenediaminetetraacetic acid, 4.5 g/L ZnSO_4_ • 7 H_2_O, 0.84 g/L MnCl_2_ • 2 H_2_O, 0.3 g/L CoCl_2_ • 6 H_2_O, 0.3 g/L CuSO_4_ • 5 H_2_O, 0.4 g/L Na_2_MoO_4_ • 2 H_2_O, 4.5 g/L CaCl_2_ • 2 H_2_O, 3 g/L FeSO_4_ • 7 H_2_O, 1 g/L H_3_BO_3_, 0.1 g/L KI. All main culture media consisted of diluted molasses as the only nutrient source. For shake flask cultivations, 0.3 M MES buffer was added. For fermentations in 150 L scale, no MES buffer was added as the pH value was controlled via the addition of pH-adjusting agent (10 M NaOH).

Molasses was added to the desired concentration of sucrose of the main culture medium. During sugar manufacturing and the storage of the molasses, the sucrose partially decomposes into the respective monomers glucose and fructose due to alkaline hydrolysis and microbial activity [20]. Therefore, the concentrations of all three sugars were used for the calculation of the henceforth called sucrose equivalents *c*_SUCEQ_ according to Eq. (1):

1 $$c_{{{\mathrm{SUCEQ}}}} = \frac{1}{{M_{{{\mathrm{SUC}}}} \, \cdot \,CM_{{{\mathrm{SUC}}}} }}\, \cdot \,\sum\nolimits_{k = 0}^{3} {c_{{{\mathrm{SUG}},k}} \, \cdot \,M_{{{\mathrm{SUG}},k}} \, \cdot \,CM_{{{\mathrm{SUG}},k}} }$$

Here, *M* [g/mol] and *CM* [mol carbon/mol] are the molar mass and the number of carbon molecules per sugar molecule in sucrose (SUC) and each sugar (SUG), respectively. The exact molasses concentration for each main culture is further specified in the respective sections. To facilitate handling on a laboratory scale, molasses was diluted with distilled water to 500 g molasses per liter stock solution (corresponding to 45 wt%) before medium preparation. Stock solutions for sucrose, molasses, NH_4_Cl, KH_2_SO_4_, MgSO_4_, and NaCl were prepared separately and sterilized by autoclaving. The pH value of the KH_2_PO_4_ solution was adjusted to 6.5 with 10 M NaOH. Stock solutions for FeSO_4_, trace elements, and MES buffer were prepared separately and sterilized via sterile filtration. The pH value of MES buffer was adjusted to 7.2 with NaOH pellets. Stock solutions of sucrose, molasses, FeSO_4,_ and trace elements were stored at 4 °C. All other components were stored at room temperature. The preparation of the media for the 150 L fermenter is described in the respective section.

### Cultivation systems and online analytics

#### Shake flasks

Precultures and main cultures in shake flasks were performed in unbaffled 250 mL flasks using the in-house built Respiratory Activity Monitoring System (RAMOS) [47, 48]. With the RAMOS device, the oxygen transfer rate (OTR) was measured non-invasively in each individual flask. A commercial version of the RAMOS device can be obtained from HiTec Zang GmbH (Herzogenrath, Germany). A modified version is available from Adolf Kühner AG (Birsfelden, Switzerland). When offline samples were needed during the cultivation, additional shake flasks without online monitoring were simultaneously incubated under the same conditions and used for sampling. Shake flasks were incubated at a temperature of 30 °C, a shaking frequency of 350 rpm, and a shaking diameter of 50 mm in a Climo-Shaker ISF1-X (Kühner AG, Birsfeld, Switzerland).

Precultures were inoculated to an optical density OD_600_ measured at a wavelength of 600 nm of 0.1 (details in section Offline Sample Analytics) from glycerol stock cell suspensions and harvested during exponential growth after approximately 16 h. Precultures had a filling volume *V*_L_ of 10 mL.

Batch and pulsed batch cultures were inoculated to an OD_600_ of 0.1 directly from the preculture. Batch cultures had a filling volume *V*_L_ of 20 mL and a sucrose equivalents concentration of 27–156 g/L from molasses. Pulsed batch cultures had an initial filling volume of 10 mL with an initial concentration of 50 g/L. Fresh molasses was subsequently provided by the pulse. In order not to dilute the buffer necessary for pH stabilization, MES buffer was also added to the pulse, which reduced the sugar concentration of the feed. Consequently, an additional 10 mL (0.3 M MES buffer, molasses equivalent to 160 g/L sucrose) were added by the pulse, resulting in 105 g/L sucrose equivalents in total.

The fed-batch process was implemented in shake flasks via continuous diffusion of the feed solution according to Habicher et al. [49]. The feed rate is thereby determined by the diffusion across the dialysis membrane separating the reservoir from the cultivation medium, which is dictated by the concentration difference [50]. The membrane has a molecular weight cut-off of 20 kDa, allowing the diffusion of most nutrients such as sugars, salts, amino acids, and peptides. The preculture was prepared and harvested as described above. For fed-batch cultivations, a second preculture had to be prepared without the secondary substrates phosphate and nitrogen to deplete the organism of potential storage molecules and to trigger the malic acid production phase through limitation. Therefore, the first preculture was centrifuged at 1780 g and 4 °C for 20 min and washed once with 9 g/L NaCl. After a second centrifugation step, the cells were resuspended to an OD_600_ of 10 in preculture medium without NH_4_Cl and KH_2_PO_4_. The second preculture was then transferred to shake flasks and cultivated for 4 h until a secondary substrate limitation was visible in the OTR. The second preculture was harvested via centrifugation. The cells were resuspended in 0.3 M MES buffer to an OD_600_ of 5 for the fed batch main culture. Molasses was not added to the initial cell suspension, but was only provided via the feed. Diluted molasses equivalent to 247 g/L sucrose was used as feed in the reservoir. The amount of sucrose, which diffused into the culture broth during the experiment, was calculated using a mass balance according to Habicher et al. [49].

#### 150 L pilot-scale fermenter

The process was scaled up to a 150 L pilot-scale fermenter unit (Proreact P ATEX, Frings, Rheinbach, Germany) equipped with a pH probe (Hamilton Polilyte Plus H VP 120) and a dissolved oxygen tension (DOT) probe (Hamilton VisiPro DO Ex 120).

The seed train for the 150 L fermentation consisted of a first preculture in shake flask scale, followed by a second preculture in a benchtop fermenter. The second preculture was conducted in a 3 L New Brunswick BioFlo/ Celligen 115 benchtop bioreactor (Eppendorf SE, Hamburg, Germany) equipped with an EasyFerm Plus PHI K8 225 pH probe (Hamilton Bonaduz AG, Bonaduz, Switzerland) and a VisiFerm DO ECS 225 DOT probe (Hamilton Bonaduz AG, Bonaduz, Switzerland). For the second preculture, 1.8 L of preculture medium was used. The addition of MES buffer was omitted, as the pH value was controlled at 6.5 by the addition of 5 M NaOH. The temperature was controlled at 30 °C and the DOT was maintained at 30% by variation of the agitation speed (300–1200 rpm). The aeration rate was set to 1 volume of air per volume of medium per minute (vvm). The fermentation was inoculated to an OD_600_ of 0.1 from the first preculture in shake flask.

For the main culture in 150 L scale, diluted molasses was used as the sole nutrient source. To mitigate foaming, antifoam 204 (Sigma-Aldrich, St. Louis, USA) was used as needed. To prevent contamination, 100 mg/L kanamycin was added. 10 M NaOH was used as pH adjusting agent during the fermentation. The fermenter was sterilized at 121 °C filled with deionized water. A concentrated molasses solution was prepared by sterilizing molasses in a 100 L feeding vessel at 121 °C under stirring and diluting to 80 wt% with deionized water. Initially, molasses was transferred from the feeding vessel to the fermenter, resulting in an initial concentration of 78 g/L sucrose equivalents (phase I, batch phase). After inoculation with 1.4 L pre-culture via septum to an OD_600_ of 0.2, the cultivation was started. The fermentation was controlled at a temperature of 30 °C, a pH value of 6.5, a DOT of 30% with an aeration rate of 1 vvm, a stirring speed between 190 and 400 rpm, and a head pressure of 0.23 bar overpressure.

The DOT and stirring speed were used as an indicator of metabolic activity, as a miscalibration of the exhaust gas analyzer resulted in erroneous OTR values. A pulsed batch operating mode was established by feeding additional molasses after the carbon source was depleted, as indicated by the metabolic activity, resulting in a replenished sugar concentration of 79 g/L (phase II) and 83 g/L (phase III). A repeated pulsed batch was established by harvesting the fermentation broth containing the dissolved product via cell biomass retention using an Al_2_O_3_ membrane (Typ 37/3.8, 0.2 μm, A-Tech, Gladbeck, Germany) at a cross-flow of 100 L/min, a permeate flow of 0.7 L/min, and a transmembrane pressure of 0.4 bar. The filling volume was reduced by 64% from 95 to 35 L, resulting in the desired concentration of the cells. Further reduction was not possible to maintain pumpability of the liquid and to ensure constant oxygen supply due to the reactor geometry. Next, the sugar concentration was replenished by a pulse of molasses to a concentration of 92 g/L sucrose equivalents (phase IV). One pulse of diluted molasses was added when sugar was depleted, resulting in a replenished sugar concentration of 86 g/L (phase V) sucrose equivalents. The fermentation broth was again harvested as described above for downstream processing. The permeate of both harvests was stored in 50 L KEG vessels at −18 °C.

To describe the fermentation performance, the KPIs fermentation yield and STY were calculated based on mass balances of the substrates sucrose, glucose, and fructose, and the product malic acid. The produced malic acid *m*_MA_ [g] and consumed sugars *m*_SUG_ [g] were calculated according to Eqs. (2)–(3):

2 $$m_{{{\mathrm{MA}}}} \, = \,c_{{{\mathrm{MA}}}} \, \cdot \,V_{{\mathrm{L}}} \, + \,\sum\nolimits_{{{\mathrm{k}} = 1}}^{n} {c_{{{\mathrm{MA}},{\mathrm{sample}},{\mathrm{k}}}} \, \cdot \,V_{{{\mathrm{sample}},{\mathrm{k}}}} }$$

3 $$m_{{{\mathrm{SUG}}}} \, = \,c_{{{\mathrm{SUG}}}} \left( {t_{0} } \right)\, \cdot \,V_{{\mathrm{L}}} \left( {t_{0} } \right) + \,\sum\nolimits_{i = 0}^{j} {c_{{{\mathrm{SUG}},{\mathrm{pulse}}}} \, \cdot \,V_{pulse,i} } \, - \,\sum\nolimits_{k = 1}^{n} {c_{{{\mathrm{SUG}},{\mathrm{sample}},{\mathrm{k}}}} } \cdot \,V_{{{\mathrm{sample}},{\mathrm{k}}}} \, - \,c_{{{\mathrm{SUG}}}} \cdot V_{{\mathrm{L}}}$$

Here, *c*_MA_ [g/L] is the concentration of malic acid, and *V*_L_ [L] is the liquid volume of the fermenter. The term ${\sum }_{k=1}^{n}{c}_{\mathrm{MA},\mathrm{sample},\mathrm{k}}\cdot {V}_{\mathrm{sample},\mathrm{k}}$ represents the malic acid mass lost due to sampling. The consumed sugars *m*_SUC_ [g] were each calculated separately. The term ${c}_{\mathrm{SUG}}\left({t}_{0}\right)\cdot {V}_{\mathrm{L}}\left({t}_{0}\right)$ represents the initial sugar mass in the medium, while the sugars fed by the molasses pulses are considered by the term ${\sum }_{i=0}^{j}{c}_{\mathrm{SUG},\mathrm{pulse}}\cdot {V}_{\mathrm{pulse},i}$. Similar to malic acid balancing, sugar loss due to sampling is considered by the term ${\sum }_{k=1}^{n}{c}_{SUG,sample,k}\cdot {V}_{sample,k}$. The resulting sucrose equivalents were calculated using Eq. (1). The fermentation yield *Y* and STY were calculated based on *m*_MA_ [g], *m*_SUCEQ_ [g], the fermentation time *t* [h], as well as the reaction volume at this timepoint *V*_L_*(t)* [L] according to Eqs. (4)–(5). For the calculation of the STY, only the fermentation time until sugar depletion was considered, as indicated by the OTR or stirring speed.

4 $$Y = \frac{{m_{{{\mathrm{MA}}}} }}{{m_{{{\mathrm{SUCEQ}}}} }}$$

5 $$STY = \frac{{m_{{{\mathrm{MA}}}} }}{{V_{{\mathrm{L}}} \left( t \right)\, \cdot \,t}}$$

### Downstream process

#### pH-shift and adsorption

To purify the cell-free fermentation broth (150 L fermenter, Phase V), pH was adjusted to pH 2 with 95% sulfuric acid from VWR International (Radnor, PA, United States) and then filtered by vacuum filtration using MW 640 w filter paper discs (Macherey–Nagel GmbH & Co. KG, Düren, Germany).

The adsorption experiments were performed using the Azura chromatography system by Knauer Wissenschaftliche Geräte GmbH (Berlin, Germany). The system comprised of a P 2.1L pump head and an inline conductivity detector. As an adsorbent, the activated carbon 110029 was purchased from Talamon GmbH (Premnitz, Germany). To exclude air from the pores, the adsorbent was stirred in a beaker filled with ethanol for at least 30 min. The suspension was then packed into the empty glass column with a diameter of 5 cm and a length of 50 cm purchased from Götec Labortechnik GmbH (Bickenbach, Germany) using the slurry method [51]. After packing, the column was washed with distilled water for ten column volumes with a volumetric flow of 40 mL/min. The packed-bed height was 37 cm.

Before each adsorption experiment, the column was washed with 1.6 L of water. Each breakthrough experiment comprised of three phases: loading with pH-adjusted fermentation broth, elution with deionized water, and elution with acetone (Merck KGaA, Darmstadt, Germany). The volume flow rate was set to 20 mL/min in each phase. In order to develop an adsorption protocol, a preliminary breakthrough experiment was performed. Because the conductivity detector does not provide any information on the composition of the eluate, the eluate from the preliminary experiment was collected as 5 mL fractions for offline HPLC analysis. The durations of the three phases are summarized in Table 1, named preliminary adsorption protocol.

**Table 1:** Preliminary and adjusted adsorption protocols

Phase	Duration of phase in the adsorption process in min	
	Preliminary adsorption protocol	Adjusted adsorption protocol
Loading	40	16
Water elution	80	24
Acetone elution	62	78

Based on the results of the preliminary breakthrough experiment, the adsorption protocol was adjusted by shortening or lengthening the duration of each phase, as shown in Table 1. Although the volume flow rate remained at 20 mL/min, as in previous experiments, no fractions were collected in these experiments. Instead, waste and product fractions were collected. The first waste fraction contained the eluate from the entire loading phase and the first 9 min of the water elution. This comprised the wash water displaced by the fermentation broth, as well as the fermentation broth displaced during elution with deionized water. The eluate from the remaining 15 min of water elution was collected as the first product fraction, which is subsequently referred to as water eluate. At the start of the acetone elution, water was displaced by acetone. Consequently, the eluate during the first 18 min of this phase was gathered in a second waste fraction. The remaining eluate was collected in the second product, which is referred to as acetone eluate. This experiment was conducted in duplicate.

The amount of malic or succinic acid that is adsorbed during loading can be calculated by approximating the integral of concentration profiles in the eluate:

6 $$m_{ads} = \dot{V}\, \cdot \, \left( {c_{0} \, \cdot \,\Delta t - \,\sum\nolimits_{n = 0}^{k} {\frac{{c_{n} + c_{n - 1} }}{2}\, \cdot \,\left( {t_{n} - t_{n - 1} } \right)} } \right)$$

$\dot{V}$ represents the volume flow rate, ${c}_{0}$ the initial acid concentrations; ${c}_{n}$ the *n*th and ${c}_{n-1}$ the *n-1*th outlet concentrations at times ${t}_{n}$ and ${t}_{n-1}$. $\Delta t$ indicates the loading interval.

#### Decolorization of the eluates

The activated carbon Colorsorb L (Jacobi Carbons AB, Kalmar, Sweden) was used to decolorize the water and acetone eluate. Before use, the activated carbon was thoroughly washed with distilled water and dried to remove dust and other impurities. In the decolorization experiments, Colorsorb L was added to 5 mL each of the water eluate and the acetone eluate from the malic acid adsorption process. The mass of activated carbon ranged from 0.1 to 0.5 g. After 24 h on an orbital shaker (Adolf Kühner AG, Birsfelden, Switzerland), the liquid phase was removed from the activated carbon and filtered through a syringe filter (CHROMAFIL Xtra H-PTFE-20/13, Macherey–Nagel GmbH & Co. KG, Düren, Germany). The UV–Vis spectra from 350 to 600 nm were recorded using a GENESYS 50 spectrophotometer (Thermo Fisher Scientific Inc., Waltham, Massachusetts, United States). Malic acid concentrations of the liquid phases were analyzed by HPLC.

By integrating the measured absorbance ${E}_{\lambda }$ from 350 to 600 nm, the absorption areas ${A}_{\mathrm{Absorption}}$ were calculated.

7 $$A_{{{\mathrm{Absorption}}}} \, = \,\int_{{350{\text{ nm}}}}^{{600{\text{ nm}}}} {E_{\lambda } }$$

Additionally, the decolorization yield was defined as

8 $$Y_{Decolorization} = \frac{{m_{{{\mathrm{CA}},{ }1}} }}{{ m_{{{\mathrm{CA}},{ }0}} }}$$

where ${m}_{\mathrm{CA}, 0}$ and ${m}_{\mathrm{CA}, 1}$ are the masses of the carboxylic acids at the start and end of the decolorization experiment, respectively. By assuming that the liquid volume of the batch decolorization experiments remains constant, Eq. (8) reduces to

9 $$Y_{Decolorization} = \frac{{c_{{{\mathrm{MA}},{ }1}} }}{{ c_{{{\mathrm{MA}},{ }0}} }}$$

where ${c}_{\mathrm{MA}, 0}$ is the malic acid concentration at the start and ${c}_{\mathrm{MA}, 1}$ at the end of the decolorization experiment.

#### Solubility of malic and succinic acid

The solubilities of malic and succinic acid were determined by adding malic acid (99%, Thermo Fisher Scientific Inc., Waltham, Massachusetts, United States) and succinic acid (99 + %, Ultrapure, Thermo Fisher Scientific Inc., Waltham, Massachusetts, United States) in excess to distilled water and acetone, respectively, to ensure saturation at equilibrium. After preparation, the samples were stirred in temperature-controlled water baths. After 24 h, the saturated supernatant was separated from the solid phase using a syringe filter, diluted, and analyzed by HPLC.

The theoretical maximum yield of a crystallization process

10 $$Y_{Crystallization} = \frac{{CF\, \cdot \,c_{{{\mathrm{MA}},0}} - c_{{{\mathrm{MA}}}}^{*} }}{{CF\, \cdot \,c_{{{\mathrm{MA}},0}} }}$$

can be estimated from the solubility limit of malic acid${c}_{\mathrm{MA}}^{*}$, the initial malic acid concentration ${c}_{\mathrm{MA},0}$, and the concentration factor $CF$ which is defined as follows

11 $$CF = \frac{{V_{0} }}{{V^{*} }}$$

where ${V}_{0}$ is the initial volume and ${V}^{*}$ is the volume at the end of the concentration step.

### Offline sample analytics

For measurements of the OD_600_, culture broth was diluted with 9 g/L NaCl solution in a range of 0.1 to 0.4 and measured with a Genesys 20 spectrophotometer (Thermo Fisher Scientific Inc., Waltham, Massachusetts, United States) using 1.6 mL Rotilabo cells (Carl Roth GmbH + Co. KG).

For the measurement of the cell dry weight (CDW), 2 mL of fermentation broth was filled into a pre-weighted reaction tube and centrifuged at 18,000 g for 10 min. The cell pellet was dried for 48 h at 80 °C and subsequently weighed.

To quantify sucrose, glucose, fructose, malic acid, and succinic acid, the samples were analyzed via HPLC. In preparation for HPLC analysis, 2 mL sample was centrifuged at 18,000 g for 5 min, followed by sterile filtration of the supernatant. The centrifugation step was omitted for cell-free samples. A Thermo Fisher Ultimate 3000 (Thermo Fisher Scientific, Waltham, Massachusetts, United States) combined with a Rezex ROA–Organic Acid H + (8%) LC Column 300 × 7.8 mm (Phenomenex, Inc., Torrance (CA), United States) and a RefractoMax 520 RI detector (Thermo Fisher Scientific, Waltham, Massachusetts, United States) was used. As a mobile phase, 25 mM sulphuric acid with a flow velocity of 0.8 mL/min at 20 °C was applied. In the applied HPLC method, all types of inorganic salt elute simultaneously as one peak. The area of this peak ${A}_{\mathrm{Salt}}$ was normalized to the salt area measured in the fermentation broth ${A}_{\mathrm{Salt},\text{ FB}}$. The normalized salt area ${A}_{\mathrm{Salt}}^{*}$ is therefore defined by the following equation:

12 $$A_{{{\mathrm{Salt}}}}^{*} = \frac{{A_{{{\mathrm{Salt}}}} }}{{A_{{{\mathrm{Salt}},\,{\mathrm{FB}}}} }}$$

Samples for the analysis of osmolality were prepared as specified for HPLC and analyzed with a Gonotec Osmomat 3000 Single-Sample Freezing Point Osmometer (Gonotec Meß- und Regeltechnik GmbH, Berlin, Germany).

## Results and discussion

In this work, a process for malic acid production from molasses was implemented based on the conceptual process design guiding fermentation and downstream development.

### Development and scale-up of the fermentation process

#### Batch process is limited by growth inhibition

**Fig. 2:**
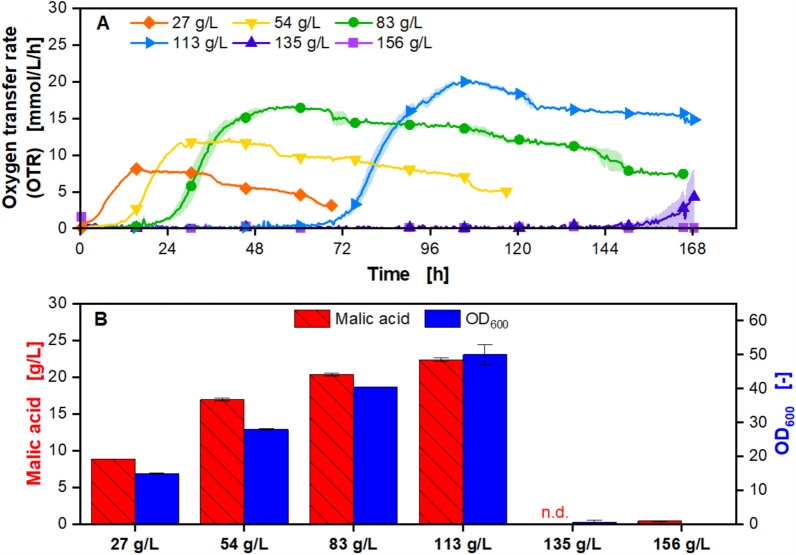
Malic acid production from molasses as a batch process in shake flasks. **A** Online data of OTR over time. For clarity, only every 30th data point is represented as a symbol. Lines are drawn through all measuring points. Shadows indicate the minimum and maximum values of biological duplicates. **B** Final malic acid concentration and OD_600_. Samples were drawn after sugar was depleted as indicated by the drop in OTR (27, 54 and 83 g/L) or after 168 h (113, 135 and 156 g/L). Error bars indicate the minimum and maximum values of biological duplicates. Cultivation was performed with *Ustilago trichophora* with an OD_600, Start_ = 0.1, and *V*_*L*_ = 20 mL in shake flasks monitored by RAMOS. The medium consisted of diluted molasses with 0.3 M MES (27–156 g/L sucrose equivalents)

Sugar beet molasses was previously identified as a viable feedstock for organic acid production with Ustilaginaceae [15, 25]. However, microbial growth inhibition by high molasses concentrations is well known in the literature and can affect product titers during batch processes [16, 52, 53]. The influence of increasing initial molasses concentrations on growth and malic acid production with *U. trichophora* was therefore investigated in shake flasks (Fig. 2).

The OTR curves show the distinct phases of secondary substrate-limited growth with a clear increase in the duration of the lag phase as molasses concentrations increase. Secondary substrate limitation is imperative for malic acid production with *U. trichophora*, and molasses lacks sufficient amounts of phosphate and nitrogen for unlimited growth [25]. The distinctive cultivation phases are described exemplarily for the cultivation on 27 g/L sucrose. A closely monitored reference cultivation on minimal medium is provided in the supplementary data (Additional file 1: Figure S1) to support the conclusions drawn from the OTR data. The initial growth phase is visible as an exponential increase in the OTR during the first 7 h. When phosphate is presumably depleted, the slope of the OTR decreases until the OTR reaches a plateau after 15 h. The OTR drops after 36 h to a lower plateau and drops again after 62 h, which indicates the depletion of glucose and fructose, respectively. During the limitation phase, malic acid is produced. With increasing molasses concentration, the concentrations of both the limiting secondary substrate and the carbon source increase. For molasses concentrations up to 113 g/L, this leads to higher biomass formation and malic acid titers. After 168 h, the highest malic acid titer of 22.4 ± 0.2 g/L was achieved for 113 g/L sucrose equivalents with a yield of 0.25 g/g, a STY of 0.13 g/L/h, and a remaining substrate concentration of 23.7 ± 0.7 g/L sucrose equivalents. Succinic acid is a precursor of malic acid in the TCA and a known byproduct of the wild-type strain *U. trichophora* [54]. Up to 5.5 g/L succinic acid was detected in the fermentation broth after the cultivation. An overview of all yields, STYs, and succinic acid titers can be found in the supplementary data for successful malic acid productions (Additional file 1: Table S1 and Table S2).

Severe substrate inhibition becomes visible with increasing molasses concentrations, which results in significantly prolonged lag phases or complete growth inhibition. Microbial growth inhibition by molasses is well-documented in the literature, and high osmolality, along with inhibitors such as lactic acid, are most commonly suspected to be the main influencing factors [16, 55, 56]. One aim of this work was to use untreated molasses to lower the overall production costs. Therefore, the detailed analysis of the influence of each impurity in molasses was omitted, but osmolality was examined exemplarily, as shown in the supplementary data (Additional file 1: Figure S2). High osmolalities indeed lead to a significant growth inhibition of the organism. However, *U. trichophora* is remarkably osmotolerant, and the observed inhibition at high molasses concentrations most likely results from both factors. Adapted laboratory evolution might be a possible approach to increase the strain’s tolerance towards molasses [24]. It also becomes evident that the possibilities for process optimization via medium optimization are very limited. When using defined minimal medium, as is the state-of-the-art, the concentration and thus the ratio of the carbon source and the secondary substrates can be adjusted separately for an optimal process [21, 57, 58]. However, when using molasses, the ratios are predetermined by the complex substrate. Supplementing the secondary substrates would accelerate the process due to higher biomass concentrations, although this would result in a lower yield [25]. A common approach in the literature to overcome the unfavorable effects of molasses is the partial replacement with purified sugar, which would result in higher final titers and product yields [59]. Although effective, this strategy contradicts the initial motivation of using residue streams.

#### Investigation of fed-batch and pulsed batch in shake flasks

**Fig. 3:**
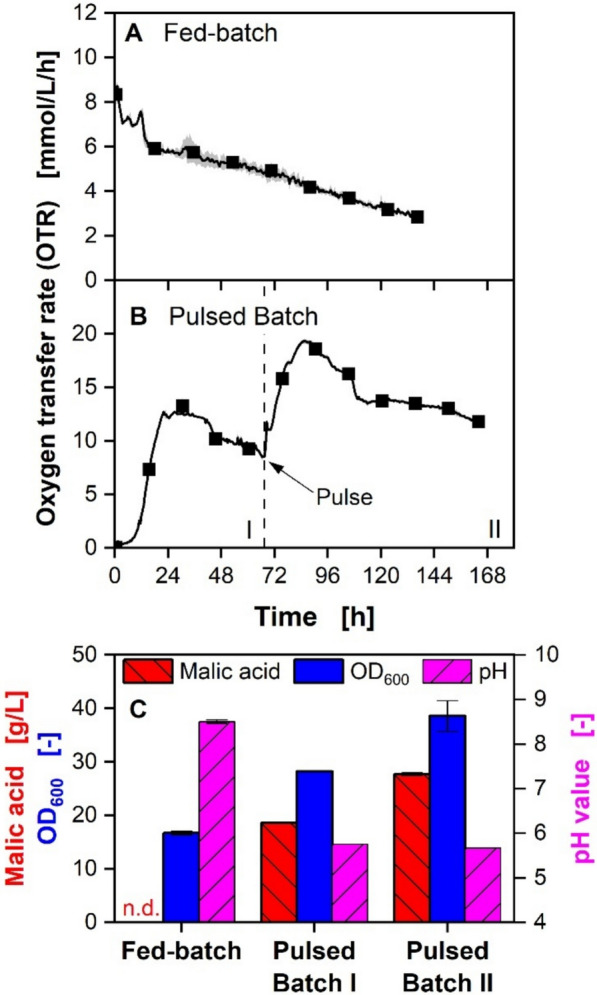
Fed-batch and pulsed feeding of molasses in shake flask for malic acid production. **A**, **B** Online data of OTR over time. For clarity, only every 30th data point is represented as a symbol. Lines are drawn through all measuring points. Shadows indicate the minimum and maximum values of biological duplicates. **C** Malic acid titer, OD_600,_ and pH value. Error bars indicate the minimum and maximum values of biological duplicates. Intermediate measurement after pulsed batch phase I was conducted as single measurement. Cultivation was performed with *Ustilago trichophora* in shake flasks monitored by RAMOS. **A** Continuous molasses feeding via diffusion over a dialysis membrane in a fed-batch flask. OD_600, Start_ = 5, *V*_*L*_ = 20 mL. The initial medium consisted of 0.3 M MES, and diluted molasses equivalent to 247 g/L sucrose was used as feed. In total, 20 g/L sucrose equivalents were added to the cultivation. **B** Pulsed feeding marked by initial batch phase (I), followed by molasses pulse (II). OD_600, Start_ = 0.1. The initial medium consisted of diluted molasses equivalent to 50 g/L sucrose and 0.3 M MES with *V*_L,I_ = 10 mL and diluted molasses equivalent to 160 g/L with 0.3 M MES were added with the pulse to *V*_L,II_ = 20 mL. In total, sucrose equivalent to 105 g/L was added to the cultivation

As the performance of the batch process is severely impaired, two feeding strategies were investigated (Fig. 3). During fed-batch fermentation, molasses is continuously added to the fermentation broth at a rate below the maximal possible consumption rate, resulting in a culture limited in both secondary substrate and carbon source. The second feeding strategy tested was pulsed feeding. First, a standard batch process is conducted using a low, non-inhibiting molasses concentration. After the carbon source is depleted, as indicated by the drop in the OTR, a pulse of additional molasses is added to the shake flask, which replenishes the substrate. Offline samples were drawn at the end of the fed-batch fermentation, as well as before the addition of the molasses pulse and at the end of the pulsed batch fermentation. The OTR curves of the precultures for the fed-batch process and the final succinic acid titers can be found in the supplementary data (Additional data 1: Table S2 and Figure S3).

The fed-batch process starts with a high initial OTR due to the high inoculation density (50 × higher than in the pulsed batch process). A decreasing OTR plateau is visible over time, showing the consumption of the carbon source provided by the molasses feed (Fig. 3A). In total, 19.7 ± 0.1 g/L sucrose equivalents were fed by the molasses feed. No residual sugar was detected in the culture broth at the end of the cultivation. The OTR trajectory is characteristic of diffusion-driven fed-batch processes. The uptake rate of the carbon source by the microorganism and hence the OTR is dictated by the diffusion rate across the membrane. The concentration gradient driving the diffusion decreases with decreasing sucrose concentration in the reservoir during the cultivation [50]. In addition, water diffuses through the membrane into the reservoirs via osmosis, further decreasing the molasses concentration in the reservoirs [49]. The OD_600_ increased from 5 to 16.7 ± 0.4, and the pH value increased from initially 7.2 to 8.5 ± 0.0 (Fig. 3C). The increase in the pH value can be attributed to the consumption of the lactic acid provided by the molasses, as no lactic acid was measurable at the end of the experiment. When present in excess, *Ustilago* sp. usually exhibit a preference for glucose over fructose over lactic acid [15, 56]. However, microorganisms can consume several carbon sources simultaneously under carbon-limited conditions [15, 60]. No malic acid or succinic acid was detected at the end of the fed-batch experiment. The experiment was repeated once and yielded reproducible results, confirming these findings (data not shown).

For phase I of the pulsed batch process, the characteristic OTR curve for batch cultivations is visible, and 18.5 g/L malic acid were produced. After the assumed depletion of the carbon source after 66 h, as indicated by the OTR, a molasses pulse was supplied to the culture to replenish the sucrose concentration. The organism resumed growth until the secondary substrates were again depleted after 85 h. After 164 h, an OD_600_ of 38.6 ± 2.9 and a malic acid titer of 27.6 ± 0.3 g/L were reached. HPLC analysis revealed that 19.1 ± 0.4 g/L fructose was still present in the culture broth at the end of the cultivation, resulting in a malic acid yield of 0.32 g/g with a STY of 0.16 g/L/h.

Molasses-based fed-batch proved to be unsuitable for malic acid production with *U. trichophora*. Organic acid production is often hypothesized as part of the overflow metabolism to spend energy when growth is limited by, e.g., secondary substrate limitation, and the carbon source is present in excess [61]. *Escherichia coli* is presumed to produce malic acid during secondary substrate limitation as a means to regenerate cofactors needed for the tricarboxylic acid cycle [62]. However, during fed-batch cultivations, supplied sugar is directly consumed by the organism and substrate excess is consequently prevented. In addition, secondary substrate limitation is imperative for malic acid production with *U. trichophora* [25]. Continuous feeding of molasses supplies secondary substrates simultaneously to the carbon source, which might suppress malic acid production.

Conversely, the pulsed batch provides the carbon source in excess during a distinctively substrate-limited production phase. A higher titer, yield, and STY were reached in the pulsed batch in comparison to the batch process. As the pulsed batch proved to be a viable solution to overcome the severe growth inhibition imposed by high initial molasses concentrations, it will be further pursued in the next section.

#### Scale up of pulsed batch to 150 L pilot-scale fermenter

**Fig. 4:**
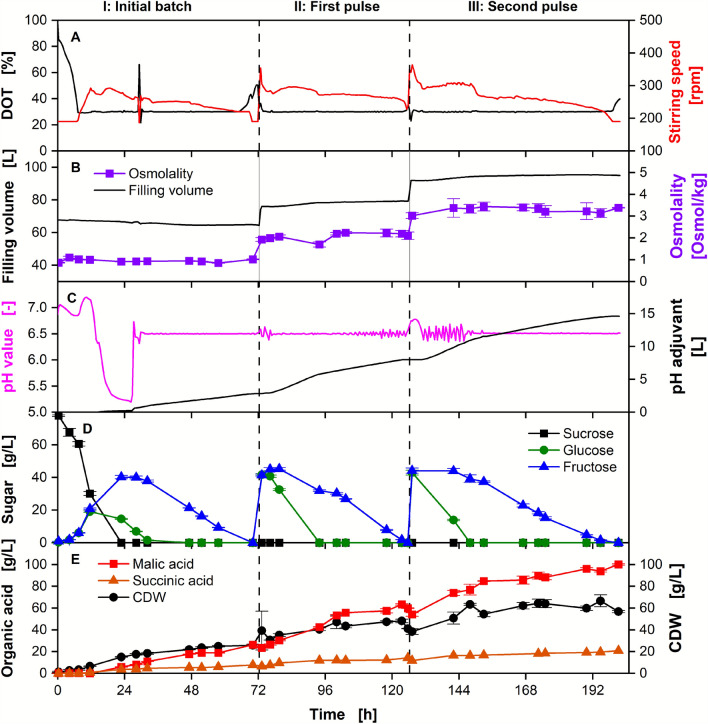
Pulsed batch fermentation for malic acid production on diluted molasses in a 150 L fermenter. Depicted are **A** DOT and stirring rate, **B** filling volume and osmolality, **C** pH value and added pH adjusting agent, **D** concentrations of sucrose, glucose, and fructose, **E** malic acid and succinic acid concentration, and CDW over time. Cultivation was performed with *Ustilago trichophora* with an OD_600, Start_ = 0.2, and *V*_*L, Initial*_ = 67 L in a 150 L fermenter. The initial medium consisted of diluted molasses (phase I: 78 g/L sucrose equivalents). Two pulses of diluted molasses were added when sugar was depleted, as indicated by a drop in the OTR, resulting in a replenished sugar concentration of 79 g/L (II) and 83 g/L (III) sucrose equivalents. In total, molasses equivalent to 198 g/L sucrose equivalents was consumed during the fermentation. For concentrations and osmolality, mean values and standard deviations of technical triplicates are shown. For continuously recorded values, a 30 min moving average is shown

To demonstrate the technical feasibility of the process for industrial application, the pulsed batch was implemented in a 150 L fermenter (Fig. 4). The fermentation process was started on diluted molasses, and two pulses were added when the sugar was depleted. No OTR data could be provided, as a miscalibration of the exhaust gas analyzer resulted in erroneous values.

The batch phase (I), first pulse (II), and second pulse (III) are evident in both online and offline data. Sucrose is hydrolyzed into its monomers glucose and fructose, a reaction likely catalyzed by an invertase common for *Ustilago* spp. [15]. The organism prefers glucose over fructose, and the depletion of the sugars is visible as two consecutive drops in the stirring speed, which is used to control the DOT to the setpoint of 30%. This online signal enables the timely addition of further substrate in the absence of OTR data. As the pH value of the medium was not adjusted during preparation, the initial pH value is above the controlled setpoint of 6.5. During the first 10 h, the pH value first decreases, then increases again, likely due to consumption of alkaline and acidic nutrients from the molasses. Unlike for the fed-batch process, where the co-consumption of lactic acid is regarded as the predominant factor, lactic acid consumption is not expected during the growth phase under sugar excess. Other nutrients, such as nitrogenous compounds, are expected to influence the pH value upon consumption [15]. *Ustilago* spp. are known for the sequential consumption of preferred nitrogen sources, which would result in the observed pattern [63]. After the onset of secondary substrate limitation, malic acid is produced, resulting in a further decrease in the pH value. Owing to the malfunctioning of the pH adjusting agent pump, the pH value decreased to 5.2 below the setpoint of 6.5. Below a pH value of 5.5, the production rate of malic acid decreases due to pH inhibition [24]. The pH control was restored afterwards. At the end of phase I, 25.9 ± 1.0 g/L biomass (CDW) and 26.3 ± 0.3 g/L malic acid were produced.

When the carbon source was depleted after approximately 65 h and 125 h, further molasses was promptly added to the fermentation broth to replenish the sugar. Metabolization of sugar and biomass growth restarted immediately after the addition of molasses, as indicated by the increase in stirring speed and CDW. No sucrose was measurable via HPLC, indicating an immediate hydrolysis into glucose and fructose. The osmolality increased with each addition of molasses and stayed approximately constant throughout each phase. In contrast to phase I, the malic acid titer supersedes the CDW. At the end of phase II, 41.2 ± 0.2 g/L CDW and 59.9 ± 0.3 g/L malic acid were measured. After phase III, at the end of the experiment, 56.8 ± 1.0 g/L CDW and a final malic acid titer of 100.1 ± 0.6 g/L were produced. The byproduct succinic acid was also detected at a final concentration of 22.9 g/L.

The addition of the pH adjuvant can be used as a real-time indicator of microbial organic acid production in pH-controlled fermentations [64, 65]. This correlation also allows for a higher time-resolved monitoring than via HPLC values. As shown in the supplementary data (Additional file 1: Figure S4), the pH adjuvant addition during the pulsed batch fermentation shown in Fig. 4 is indeed linearly proportional to malic acid production. The pH adjuvant addition and consequently malic acid production rate are significantly higher when both sugars are present compared to after glucose depletion (2.4-fold in phase II, 2.3-fold in phase III), meaning that *U. trichophora* is able to use glucose more efficiently than fructose [15]. This can likely be attributed to the higher uptake rate of glucose compared to fructose, as is visible from the HPLC measurements. In addition, a slow decrease in the pH-adjusting agent addition rate is visible at the end of the fermentation. This might be attributed to product inhibition, a common challenge of organic acid production [21, 43]. It is therefore hypothesized that the addition of further molasses will not further increase the product titer. Notably, large amounts of pH adjustment agent are added during the process, which will constitute a significant share of the process costs [9]. Other strains, such as the engineered malic acid producer *S. cerevisiae* or the itaconic acid producer *U. cynodontis,* are capable of producing at lower pH values of 2.3 and 3.6, with no or significantly reduced pH adjuvant usage, respectively [9, 66]. Future work should be dedicated to increasing the tolerance of *U. trichophora* towards low pH values, for example via laboratory adaptive evolution [66]. This would result in a strain combining the benefits of high osmotolerance and low pH adjuvant usage.

Cumulatively, molasses equivalent to 198 g/L sucrose equivalents were consumed by the organism. As previously shown, this concentration would have completely inhibited growth in a batch process. Pulsed batch proved to circumvent limitations imposed by the use of molasses as the sole carbon source for malic acid production with *U. trichophora*. Interestingly, no lag phase was observed for this experiment, which is in contrast to the batch cultivations in shake flask scale. This can presumably be attributed to the lower osmolality, as no buffer had to be added in fermenter scale. Initially, an influence of the buffer on the process was not expected based on previous results [25]. Unfortunately, this observation impedes the transferability of the results between the scales. It is therefore suspected that the initial molasses concentration could have been increased even further, which would be beneficial for the process. However, the osmolality at the end of the pulsed batch fermentation (phase III, 3.4 Osmol/kg) still exceeded the highest osmolality of the batch experiment (2.5 Osmol/kg). This further emphasizes the necessity of the pulsed batch mode.

In total, 9.5 kg malic acid were produced from 18.8 kg sucrose equivalents, resulting in a yield of 0.51 g malic acid per g sucrose equivalents with a STY of 0.50 g/L/h. If the organism is using the oxidative TCA cycle, the experimentally achieved yield corresponds to 65% of the theoretical maximal yield. As molasses naturally contains high amounts of nitrogen and phosphate, a high proportion of the provided sugar is directed to biomass production instead of product formation. During this experiment, 43% and 33% (Cmol/Cmol) of sucrose equivalents provided were converted to malic acid and biomass, respectively. The previously published biomass composition was used for the calculation [25]. Unfortunately, a complete carbon balance could not be provided owing to the missing carbon dioxide transfer rate. Furthermore, the complex feedstock molasses can obtain up to 5% further unquantified carbon sources (relative to its dry weight) [15].

The time of substrate addition was previously determined directly from the online data (either OTR or DOT and stirring speed). However, even with a fast reaction, a period of sugar depletion cannot be avoided, which reduces the STY. Additionally, this may lead to changes in the microorganism’s metabolism, which might negatively influence further productivity after the sugar has been replenished [67, 68]. Direct monitoring of the sugar concentration might offer an improvement, whereby new sugar would be added before it is fully consumed. However, established methods suffer from other disadvantages: HPLC samples require a higher manual workload, and the results are usually time-delayed, and spectroscopic or photometric methods are often impeded by the high fluorescence and coloration of the molasses [9, 69, 70]. The predictive correlation of oxygen consumption with sugar consumption is especially challenged by the complex composition and batch-to-batch variability of molasses, resulting in stoichiometric coefficient variations [67]. As the metabolic activity of *U. trichophora* was restored immediately after feeding, the cells were able to cope with short periods of nutrient depletion. The procedure shown here is therefore particularly robust and easy to implement. However, future process optimization should focus on the implementation of automatic controls or predictive models.

The long process time of 200 h remains a challenge for microbial malic acid production on molasses. Generally, processes under secondary substrate limitation are performed in two steps: growth and production phase. When feeding pure sugar, no further biomass is produced during the production phase. Molasses feeding results in a constant production of further biomass, resulting in both comparatively low production rates at the beginning and an overall low yield. However, the continuous supply of new secondary substrate has the advantage of maintaining cell viability and productivity better than when using pure sugars [43]. To improve the productivity of the process, the biomass can be recycled for the next fermentation, to circumvent a de novo phase of low productivity. This approach has already been proposed in the past by Hosseinpour Tehrani et al*.*, but could previously not be established satisfactorily due to limitations dictated by the lab-scale equipment [71]. In the following, a cross-flow tubular ceramic membrane module was used to conduct a repeated pulsed batch with cell retention.

#### Repeated pulsed batch with cell retention increases productivity

**Fig. 5:**
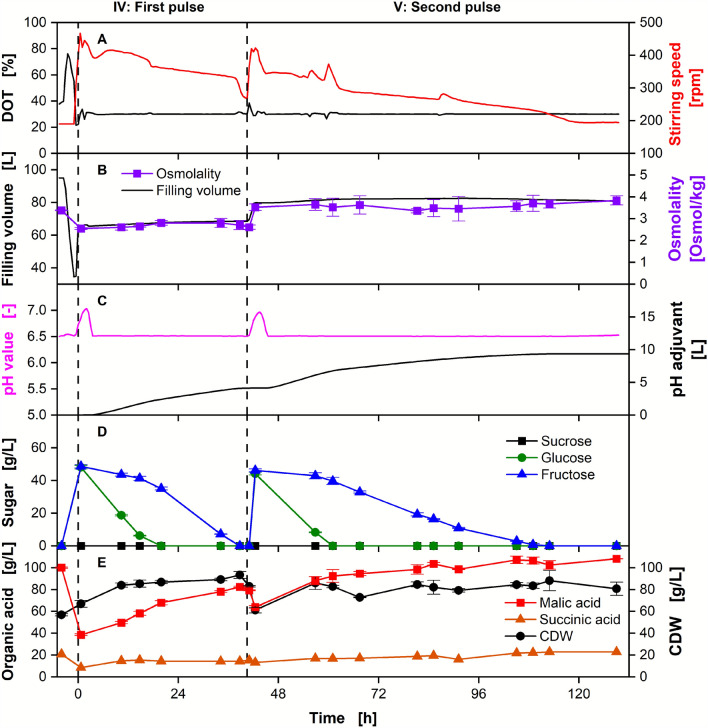
Repeated pulsed batch fermentation with cell retention for malic acid production on diluted molasses. Depicted are **A** DOT and stirring rate, **B** filling volume and osmolality, **C** pH value and added pH adjuvant, **D** concentrations of sucrose, glucose, and fructose, **E** malic acid and succinic acid concentration, and CDW over time. Cultivation was performed with *Ustilago trichophora* in a 150 L fermenter. Subsequent to the cultivation in Fig. 4, cultivation broth was first removed via a membrane with cell biomass retention, and sucrose was replenished to 92 g/L via a molasses pulse (IV). One pulse of diluted molasses was added when sugar was depleted as indicated by a drop in the OTR, resulting in a replenished sugar concentration of 86 g/L (V) sucrose equivalents. In total, molasses equivalent to 159 g/L sucrose equivalents was consumed during the fermentation. For concentrations and osmolality, mean values and standard deviations of technical triplicates are shown. For continuously recorded values, a 30 min moving average is shown

The fermentation broth was partially harvested while retaining the biomass via the membrane, and the sugar was replenished. During harvesting, 7.0 kg of malic acid were removed from the fermenter, with 2.5 kg of malic acid remaining in the vessel. The data from the repeated pulsed batch is depicted in Fig. 5.

By diluting the fermentation broth with fresh feed, the malic acid titer could be decreased from 100.06 ± 0.6 to 38.6 ± 0.8 g/L while increasing the CDW from 56.8 ± 1.0 to 66.9 ± 3.1 g/L. Biomass growth and malic acid production restarted afterwards without a visible lag phase, meaning that the cells were not significantly affected by the membrane separation. The assumed product inhibition was relieved by the malic acid dilution and is not detectable in the consumption rate of the pH-adjusting agent during phase IV. However, when the malic acid titer is again surpassing 100 g/L, production is severely inhibited, which significantly extends the duration of phase V. At the end of the fermentation, 80.8 ± 6.0 g/L CDW, 108.1 ± 0.1 g/L malic acid, and 22.9 g/L succinic acid were measured. Molasses equivalent to 159 g/L sucrose was consumed in total. During the repeated pulsed batch, 6.2 kg malic acid were produced from 12.8 kg sucrose equivalents, which resulted in an overall yield of 0.48 g/g and a STY of 0.70 g/L/h. With the additional 2.5 kg malic acid already present in the fermentation broth at the start of phase IV from the previous cultivation, 8.7 kg malic acid could be harvested at the end of phase V.

**Fig. 6:**
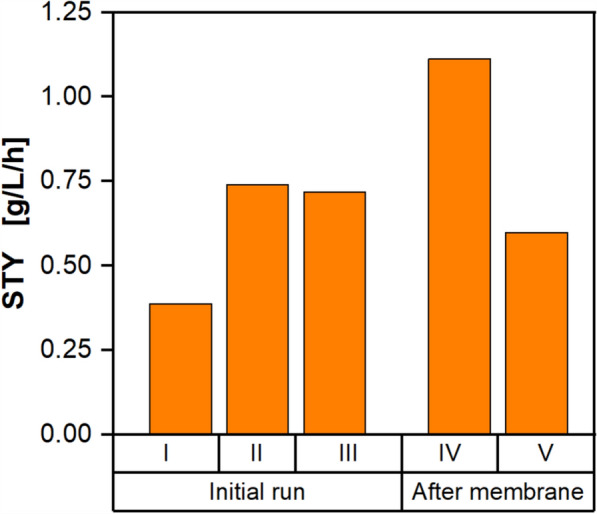
STYs of phases I-V of the repeated pulsed batch fermentation with cell retention. Phases I-III denote the initial pulsed batch fermentation, whereas phases IV-V belong to the cultivation after partial harvest with cell retention. Cultivation conditions are given in Figs. 4 and 5

To evaluate the hypothesized increase in productivity, an overview of the STYs of phases I–V of both fermentations is given in Fig. 6.

Biomass concentration and product inhibition were identified as the main factors affecting process productivity. The positive influence of higher biomass concentrations is clearly visible in the increased STYs of the later phases II-V compared to phase I. On the other hand, the prolonged cultivation time due to product inhibition negatively impacted the STYs of phases III and V. The highest STY was reached for phase IV, directly after the partial harvest with cell retention, combining high cell densities with a low product titer. Consequently, the repeated pulsed batch with cell recycling proved to be a viable solution to circumvent product inhibition in later stages of the fermentation while increasing the STY of the process. A more detailed investigation is needed to assess how many repeated batch cycles are feasible. The specific productivity per cell decreased over the course of the fermentation, and each new pulse replenishes nutrients. Consequently, a bleed stream has to be implemented to manage biomass accumulation.

In addition to the 15.7 kg malic acid produced through phases I-V, 6.5 kg of microbial biomass (CDW) could be harvested at the end of the repeated pulsed batch (phase V). According to the previously published biomass composition of phosphate-limited *U. trichophora*, approximately 37–43% of cellular biomass consists of proteins [25, 72], reaching the recommended protein content for fodder yeast [73]. The current main use of the feedstock molasses is as animal feed, whereby the primary value lies in the high sugar content [74]. The dual use of this process presents a promising approach to optimize resource efficiency by simultaneously producing the platform chemical malic acid while enhancing the nutritional profile of the residue stream for animal feed.

### Development of the downstream process

#### Adsorption of malic acid

After establishing a fermentation strategy with satisfactory KPIs, the fermentation broth underwent downstream processing. Following cell removal and acidification to pH 2, the fermentation broth contained malic acid and succinic acid, the by-product produced by *U. trichophora*. Furthermore, the broth showed high concentrations of inorganic salts derived from the molasses feed, the addition of base during fermentation, and the use of sulfuric acid in the pH shift. The molasses feed imparted a strong color to the broth, and minor amounts of pigments produced by *U. trichophora* further contributed to the discoloration [75].

**Fig. 7:**
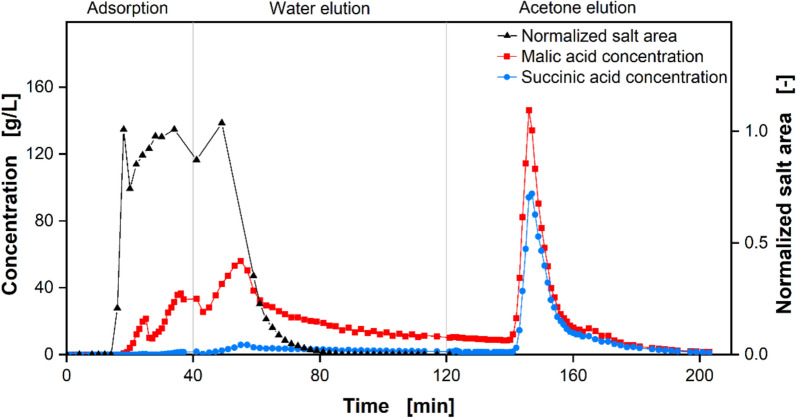
Concentration profiles of preliminary adsorption protocol. Depicted are the malic acid (red, left axis) and succinic acid concentrations (blue, left axis) as well as the profile of the normalized salt area (black, right axis)

According to the conceptual process design, an adsorption on activated carbon has been chosen as the first capture step. The breakthrough and elution profiles of all the relevant components were first determined by conducting an adsorption experiment according to the preliminary adsorption protocol. Figure 7 shows the results of this experiment, presenting the concentration profiles of malic and succinic acid, as well as the normalized salt area, over time.

Before the start of the experiment, the adsorption material was equilibrated with deionized water. By pumping the fermentation broth into the column, the adsorption process was started. After 15 min, the breakthrough of salts was detected (Fig. 7). Owing to their ionic properties, there is little to no interaction between the salt ions and the largely non-polar surface of Talamon 110029. Their breakthrough indicates the pore volume and, consequently, the total porosity of the column. The dips in the rising salt area, but also in the acid concentrations, were caused by the pump being stopped and restarted. Unlike the salts, both malic and succinic acid were retained by adsorption on the activated carbon until the capacity of the adsorbents is reached, as evidenced by their later breakthrough. In the case of malic acid, the breakthrough was already detected after 20 min, shortly after the salt breakthrough. Succinic acid, on the other hand, appeared to adsorb more strongly to the activated carbon as indicated by its later breakthrough. Although both malic and succinic acids are C4 dicarboxylic acids, malic acid is more polar than succinic acid due to the hydroxy group [76]. This results in stronger interactions of succinic acid and the non-polar carbon surface than between malic acid and the carbon surfaces. Consequently, the maximum capacity for succinic acid is higher than for malic acid. This, combined with the lower initial feed concentration, results in the breakthrough of succinic acid at 35 min. Overall, the column retains 73 wt% of malic acid and 96 wt% of succinic acid, fed during loading either through adsorption onto the carbon surface or by remaining within the interstices and pores of the adsorbent particles. After 40 min, the feed was switched to deionized water. The fermentation broth including unadsorbed malic and succinic acid was quickly washed out, as indicated by the decrease in the normalized salt area. However, following a sharp decrease in the malic and succinic acid concentrations in the eluate between 55 and 65 min, the slope in the malic acid concentration decreased considerably. This indicates a slow elution of the acid by water as malic acid desorbs and readsorbs constantly on its way through the column. Owing to the polar properties of malic acid, water is more likely to desorb this acid than succinic acid. This is also reflected in the proportion of acids eluted during water elution relative to the total mass adsorbed. Although 53 wt% of the adsorbed malic acid eluates, only 20 wt% of the adsorbed succinic acid does so. This could enable a selective desorption of malic acid. In addition, the amount of salt in the product eluate is significantly reduced as only the eluate contains the salt ions, which are washed out at the beginning of the water elution. Finally, the feed was switched to acetone, which desorbs malic acid and succinic acid equally. It interacts even more strongly with the carbon surface than the carboxylic acids, displacing both acids from the adsorption sites. This leads to an over-concentration in the outlet, with concentrations of about 150 g/L malic acid and 100 g/L succinic acid in the peak being even higher than in the feed. The acetone eluate is, in comparison to the water eluate, completely salt-free.

Based on the results, the adsorption protocol was adapted as shown in Table 1. The loading period was reduced to minimize product loss due to acid breakthrough. The duration of both the water and acetone elutions was also reduced to prevent the product solution from being diluted. The results of the optimized process are shown in Table 2. Pure malic acid was eluted in both runs carried out during the water elution. However, the malic acid concentration was only between 5 and 10 g/L. The subsequent acetone elution yielded a higher malic acid concentration of about 20 g/L, but with a purity of only around 80%, with the rest being succinic acid. The total malic acid yield of both runs was approximately 0.9.

**Table 2:** Key performance data of the optimized adsorption process

No	Water elution		Acetone elution		Total malic acid yield
	Malic acid concentration in g/L	Malic acid purity	Malic acid concentration in g/L	Malic acid purity	
1	8.22	1	21.83	0.76	0.90
2	5.46	1	23.43	0.80	0.88

#### Decolorization of the eluates

**Fig. 8:**
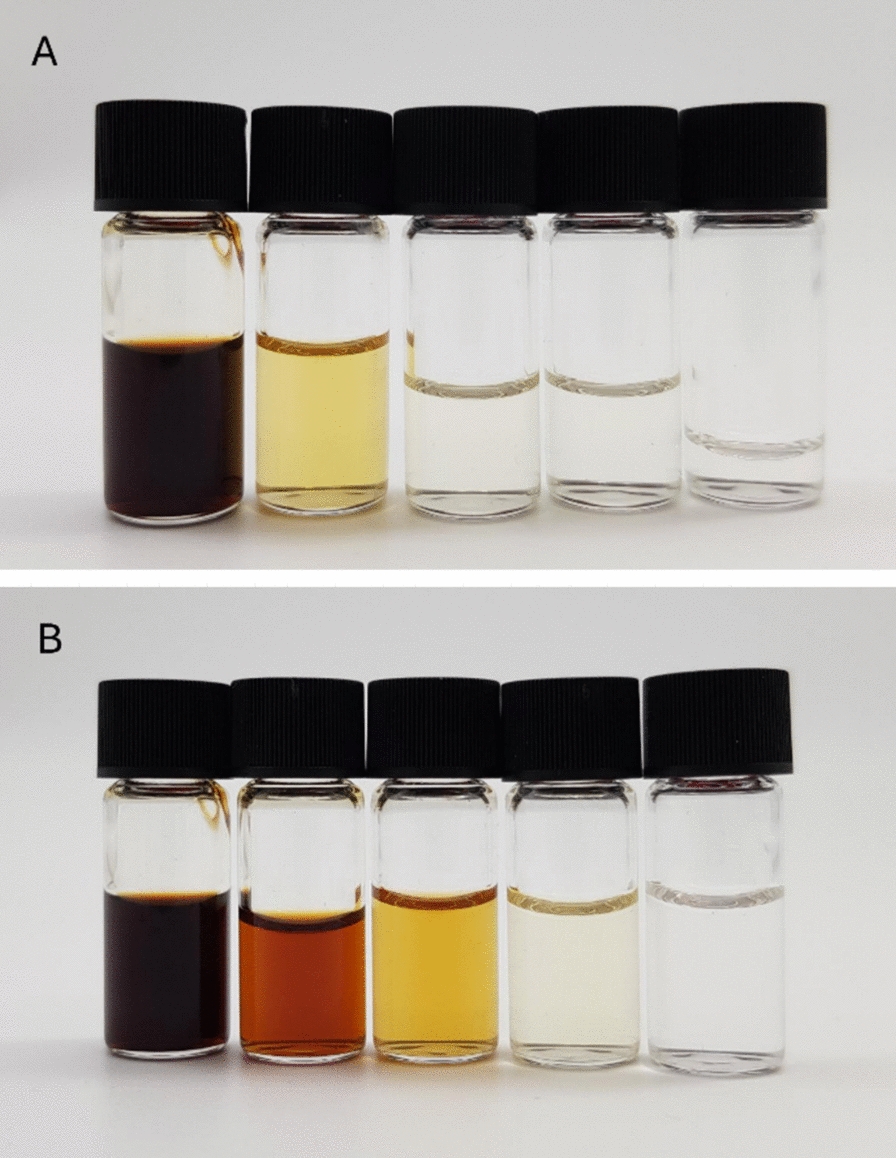
Photos of the decolorized eluates. Depicted are the decolorization of **A** acetone and **B** water; **A, B** From left to right: Fermentation broth adjusted to pH 2, eluate, decolorized solutions (20 g/L, 40 g/L, and 60 g/L activated carbon Colorsorb L)

The untreated fermentation broth, which was merely adjusted to a pH of 2, has a deep brown color, similar to molasses (Fig. 8A and B, sample on the right). During the loading phase, a large proportion of the pigments were not retained by the non-polar surface of the activated carbon Talamon 110029. As a result, the acetone and water eluates retained some color but were significantly less pigmented than the fermentation broth. The water eluate appeared amber-colored (Fig. 8B, second sample on the left), and the acetone eluate had a pale yellow color (Fig. 8A, second sample at the left). Since the aim was to produce a colorless product, the eluates required further decolorization. Unlike Talamon 110029 used in the capture step, the decolorization carbon, Colorsorb L, bears surface functional groups that interact preferentially with pigments, enabling selective color removal.

**Fig. 9:**
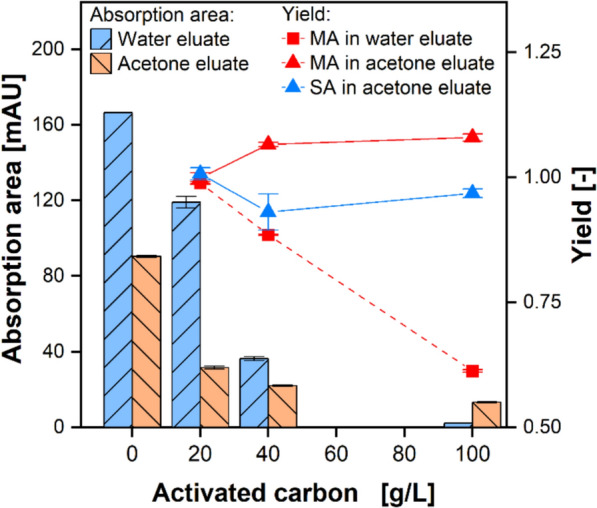
Decolorization of eluates. Left axis: Absorption areas after the decolorization of the water eluate (blue bar) and the acetone eluate (orange bar) were measured from UV–Vis spectra. Right axis: Malic acid (red) and succinic acid (blue) yield after the decolorization of the water eluate (squares) and the acetone eluate (triangles) with the activated carbon Colorsorb L are plotted over the activated carbon concentration

In addition to achieving a high degree of decolorization, the product loss due to adsorption should be minimized. The minimum amount of activated carbon required for decolorization was determined by varying the amount added to the water and acetone eluates. The resulting decolorized solutions are depicted in Fig. 8. To quantify the degree of decolorization, the absorption area for each sample was calculated using Eq. (7). Figure 9 shows the absorption area, as well as the yields of malic and succinic acid calculated using Eq. (9) for the decolorization of the water and acetone eluates against the mass concentration of the added activated carbon. Initially, the water eluate was more pigmented than the acetone eluate because pigments remaining in the pores and interstices of the adsorbent after the loading step were washed out during water elution. Consequently, roughly twice as much Colorsorb L was required for the water eluate as for the acetone eluate, for which 20 g/L of activated carbon sufficed to achieve near-complete decolorization. During decolorization of the water eluate, both malic acid and pigments adsorbed onto Colorsorb L, resulting in a 20 wt% loss at 40 g/L activated carbon. The yield of succinic acid is not specified as it is not present in the water eluate. In contrast, the malic acid yield exceeded one during the decolorization of the acetone eluate. This can be explained by the selective adsorption of acetone and pigments onto the initially unwetted carbon surface. Malic acid remained largely in solution owing to its polarity and its lower concentration relative to acetone. Consequently, the apparent equilibrium concentration of malic acid increased because the adsorbed acetone was no longer available to dilute it. Succinic acid showed the same trend as malic acid. Therefore, choosing activated carbon with a high degree of functionalization enables selective decolorization with no product loss.

#### Malic and succinic acid solubility

**Fig. 10:**
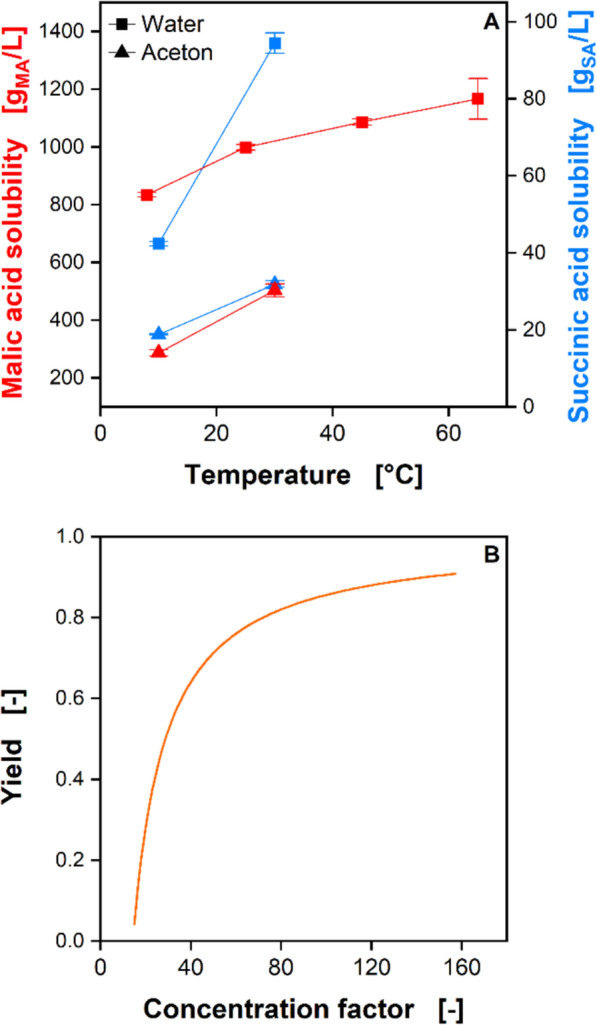
Solubility of malic and succinic acid. **A** Temperature-dependent solubility of malic acid and succinic acid in water and acetone; **B** Calculated maximum yield for the crystallization of malic acid from acetone with a start concentration of 20 g/L at a crystallization temperature of 10 °C

To obtain the final product, the crystallization of the carboxylic acids is needed. Solubility governs the theoretical maximum crystallization yield and controls selectivity. Hence, the temperature-dependent solubilities of malic and succinic acids were measured in pure component model solutions and are shown in Fig. 10A. As water is more polar than acetone, the solubilities of both carboxylic acids are significantly higher in water. In both solvents, malic acid is more soluble than succinic acid due to its greater polarity caused by the hydroxy group.

#### Selection of the eluent

As it is not possible to fully separate malic and succinic acid using adsorption alone, a second separation unit is required for further separation of the carboxylic acids. However, the experimental results indicate that adsorption on activated carbon followed directly by desorption with acetone is well-suited as an initial capture step. This method not only facilitates the early removal of impurities, such as inorganic salts, but also enables a solvent exchange from water to acetone, thereby simplifying subsequent downstream processing. Another advantage of this solvent change is that there is no product loss during the decolorization. Additionally, crystallization from acetone proves more favorable than from water due to the lower solubility of carboxylic acids in acetone. Less acetone needs to be evaporated to achieve the same crystallization yield compared with water. Furthermore, the evaporation requires less energy, since acetone has an evaporation enthalpy of 29.10 kJ/mol, whereas water has an evaporation enthalpy of 40.65 kJ/mol at atmospheric pressure [3]. Acetone also offers a selectivity advantage. Succinic acid crystallizes first from acetone because of its lower solubility, allowing for its removal prior to malic acid crystallization. However, it is important to note that a residual solubility will always remain due to solubility equilibrium; thus, achieving lower equilibrium solubility can lead to higher purity levels of the final product, malic acid. To ensure effective separation and prevent issues during processing when using acetone for desorption, it is crucial to implement a small wash step with water. This step helps remove any remaining salts and prevents direct mixing between the fermentation broth and acetone; such mixing could cause malic acid precipitation and potentially block columns and pumps.

#### Techno-economic analysis

Based on the experimental data, the process flowsheet proposed in Fig. 1 was implemented in Aspen Plus V11 (Aspen Technology, Inc., Bedford). This model is used to calculate the overall process yield and the final product purity. This serves as the basis for estimating the operating costs of malic acid production. Details of the Aspen simulation are provided in the supplementary material. In the fermentation, yields of approximately 0.5 g malic acid and 0.15 g succinic acid per g sucrose equivalents are achieved. Following pH shift and capture from the fermentation broth on activated carbon, water elution is omitted, and the two carboxylic acids are eluted simultaneously with pure acetone. In the resulting eluate, malic and succinic acid have a concentration of 20 g/L and 5 g/L, respectively. Adsorption yields are 0.90 for malic acid and 0.98 for succinic acid, according to the experimental adsorption results. During decolorization, neither malic nor succinic acid adsorb; therefore, the yields for both acids are 1.00. During evaporation, the concentration of malic acid is increased to 262.5 g/L at elevated temperatures, just below the solubility equilibrium concentration of 287.39 g/L. Cooling to 10 °C causes only succinic acid to exceed its solubility limit and crystallize as a pure substance. The total succinic acid recovery amounts to 0.10 g per g sucrose. Succinic acid can be sold as a secondary product. To crystallize malic acid, the eluate is further concentrated and cooled again to 10 °C. The crystallization yield depends on the concentration factor. Figure 10B shows the theoretical maximum yield for crystallizing malic acid from pure acetone with an initial concentration of 20 g/L at 10 °C as a function of the concentration factor. The theoretical maximum yield increases with concentration factor, reaching approximately 0.90 at a factor of approximately 144. At a factor of 44, the maximum achievable yield is approximately 0.67. This yield is sufficient for subsequent calculations because further evaporation would significantly increase energy demand. The resulting product crystals contain 97.9% malic acid and 2.1% succinic acid, attributable to the residual solubility of succinic acid. To minimize product loss, the mother liquor is recycled. Under these conditions, the total process yield is 0.43 g product per g sucrose.

**Fig. 11:**
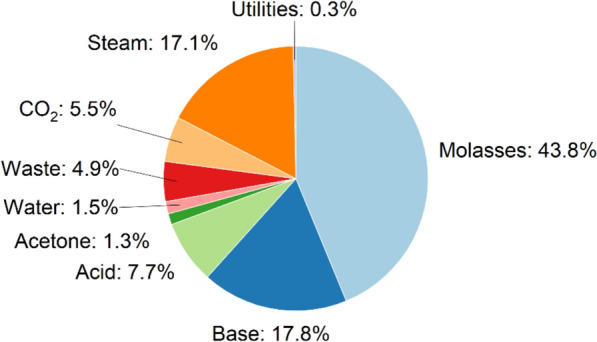
Breakdown of operating costs of malic acid and succinic acid production

On this basis, the composition of the operating costs is estimated; prices for each stream and utility are summarized in Additional file 1: Table S4. Considering both crystallized succinic and malic acid as products, the overall production cost is estimated at 1,152 EUR/t, which falls within the range of the current market price of approximately 1,250 EUR/t [77]. Figure 11 summarizes the breakdown of the operational costs. The largest cost drivers are the substrate molasses (43.8%), base for pH control during the fermentation (17.8%), and steam for acetone evaporation during the crystallization (17.1%). Reducing these costs offers substantial leverage for improvement.

Substrate costs can be lowered by increasing the substrate yield in the fermentation. The KPIs achieved in this work compare satisfactorily with studies using residue streams and demonstrate the technical feasibility of using untreated sugar beet molasses at 150 L pilot scale, which is an important step towards the utilization of residue streams. However, the KPIs do not meet the performance levels observed with highly engineered strains using pure glucose as a substrate (up to 233 g/L, 0.94 g/g, and 1.86 g/L/h) [66, 78]. Strain optimization of the wild-type *U. trichophora* is expected to have the most significant impact on process performance by improving substrate yield and thereby, the overall economics. Therefore, the following targets are proposed:

- Introduction of CO_2_ co-consumption to maximize the theoretical yield [78, 79]
- Improved tolerance towards high initial molasses concentrations [80]
- Increased tolerance towards high product titers to alleviate product inhibition
- Enhanced product formation at low pH values to reduce pH-adjuvant usage and increase compatibility with downstream processing or enable in-situ product recovery [66, 78]
- Reduced byproduct formation to enhance the production of the target product and facilitate DSP [81, 82]

*U. trichophora* currently produces malic acid only at near-neutral pH, necessitating continuous base addition to maintain a constant pH. Downstream processing, however, requires the protonated carboxylic acid, prompting the need for a pH shift. Enhancing product formation at low pH values would eliminate the need for a pH shift, reducing the amount of pH adjuvant used during fermentation and the pH shift, and improving the economics of the process. Furthermore, the compatibility with DSP is increased, or even in-situ product recovery may be enabled [66, 78].

Steam is used to evaporate acetone to reach the solubility limits of succinic and malic acid. Although switching from water to acetone has already reduced the evaporation duty, the requirement remains substantial. Further optimization of the adsorption protocol can increase the malic acid concentration in the acetone eluate and thereby reduce subsequent evaporation duty. Specifically, by collecting only the peak of the acid concentration profile (Fig. 7 at 150 min), dilution of the acetone eluate is limited, and thus the acid concentrations in the eluate increased. At this point, malic acid and succinic acid are not yet fully desorbed, reducing the per-cycle adsorption yield. However, the product is not lost, as the remaining acids are desorbed in subsequent cycles.

Overall, this study demonstrates the feasibility of the process and identifies optimization pathways that could further reduce operating costs and narrow the performance gap to petrochemical benchmarks. This would advance the industrial deployment of residue-stream-based organic acid production.

## Conclusion

Complex feedstocks introduce challenges for the fermentation and downstream process of microbial carboxylic acid production, which prohibit the direct transfer of established process strategies. In this work, a comprehensive process was tailored specifically to malic acid production from untreated sugar beet molasses to overcome common limitations. During the fermentation, substrate inhibition and a predetermined nutrient composition offer little room for maneuver in process development. The smut fungus *U. trichophora*, which was chosen due to its high osmotolerance and wide substrate spectrum, showed significant growth inhibition for molasses concentrations above 100 g/L sucrose equivalents in batch processes. Carbon excess and secondary substrate-limited conditions were identified as prerequisites for an efficient molasses feeding process. A repeated pulsed batch with membrane-based cell retention was developed in a 150 L pilot scale, with a medium consisting solely of diluted untreated molasses. A final product titer of 108.1 ± 0.1 g/L malic acid was achieved with an overall process yield of 0.50 g malic acid per g sucrose equivalents, an overall process STY of 0.66 g/L/h, and a maximal STY of 1.1 g/L/h. In total, 15.7 kg of malic acid were produced during cultivation. Additionally, the byproduct succinic acid was detected in concentrations of up to 22.9 g/L.

High pigmentation and high salt load in the fermentation broth pose significant challenges for the downstream process. To address these, an adsorption process on activated carbon was developed to capture malic and succinic acid from the broth while effectively removing impurities and performing a solvent change to acetone. The use of acetone offers several advantages in the subsequent downstream: it enables further decolorization without product loss, which is a common issue with aqueous solutions, and significantly reduces the solubility of the carboxylic acids compared to water.

Based on the experimental results, a process model was implemented in Aspen. A two-step crystallization sequence produces malic and succinic acids with purities of 98% and 100%, respectively. Overall yields of approximately 0.43 g malic acid per g sucrose and 0.10 g succinic acid per g sucrose are achieved. This approach enhances product purity, maximizes overall yields, and minimizes losses during decolorization and crystallization. A techno-economic analysis indicates process feasibility of fermentative malic acid production from untreated molasses, with production costs falling within the range of the current market price. The combination of an optimized fermentation protocol and tailored downstream processing has enabled the overcoming of typical challenges associated with utilizing agricultural residue streams.

## Supplementary Information

Additional file 1.

## Data Availability

The datasets supporting the conclusions of this article are included within the article and its additional file (Additional file 1.pdf: Figure S1, Table S1, Table S2, Figure S2, Figure S3, Figure S4, Figure S5, Figure S6, Figure S7, Figure S8, Table S3, Table S4)

## Abbreviations

HPLC: High performance liquid chromatography
DOT: Dissolved oxygen tension
OD_600_: Optical density measured at a wavelength of 600 nm
vvm: Volume of air per volume of medium per minute
STY: Space–time yield
KPI: Key performance indicator
MES: 2-(N-morpholino)ethanesulfonic acid
OTR: Oxygen transfer rate
CDW: Cell dry weight
*V*_L_: Filling volume
n.d.: Not detectable
