# Supplementary material for: From beet molasses to malic acid: holistic development of fermentation and downstream process
Source: Biotechnol Biofuels Bioprod. 2026 Feb 3;19:24. doi: 10.1186/s13068-026-02736-8 (PMC12930559; doi:10.1186/s13068-026-02736-8)
Supplement: Supplementary file 1 — Additional file 1. [file 13068_2026_2736_MOESM1_ESM.pdf]

**Additional file 1**

**From beet molasses to malic acid: Holistic development of fermentation and downstream process**

Luca Antonia Grebe[1, †], Christina Krekel[2, †], Constantin Alexander Maaß[1], Mario Beckers[3], Martin Smotrycki[3], An N.T. Phan[4], Lars M. Blank[4,5], Katharina Saur[2], Marcel Mann[1], Jörn Viell[3], Andreas Jupke[2,6]*, Jørgen Barsett Magnus[1,5]*

[†]Authors contributed equally to this work

[1]AVT.BioVT - Biochemical Engineering, RWTH Aachen University, Forckenbeckstraße 51, 52074 Aachen, Germany

[2]AVT.FVT - Fluid Process Engineering, RWTH Aachen University, Forckenbeckstraße 51, 52074 Aachen, Germany

[3]AVT – NGP[2] Biorefinery, RWTH Aachen University, Forckenbeckstraße 51, 52074 Aachen, Germany

[4]Institute of Applied Microbiology-iAMB, Aachen Biology and Biotechnology-ABBt, RWTH Aachen University, Worringer Weg 1, 52074 Aachen, Germany

[5]WSS Research centre „catalaix“, Germany

[6]IBG-2 – Institute for Bio- and Geosciences, Forschungszentrum Jülich GmbH, Johnenstraße, 52428 Jülich, Germany

## Fermentation development

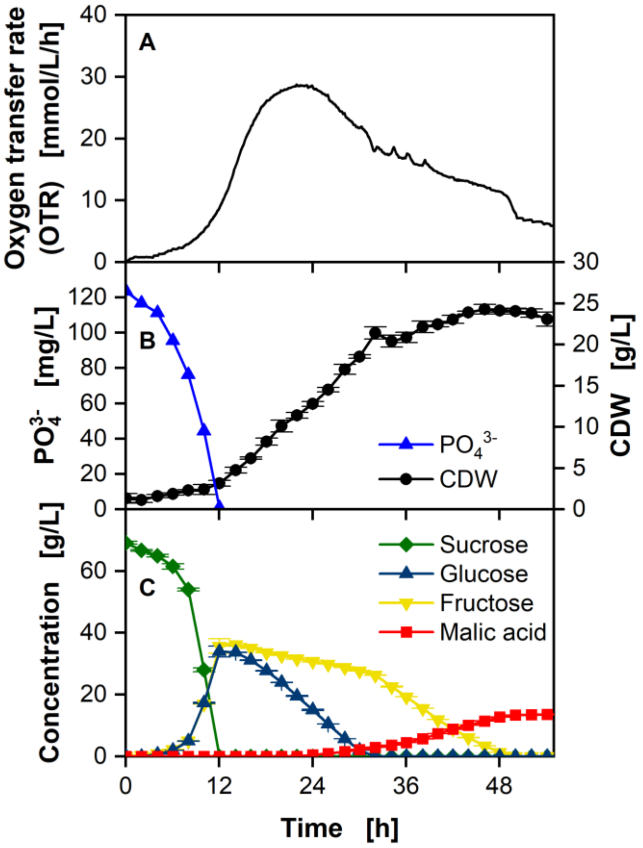


**Figure S 1: Reference cultivation of phosphate-limited malic acid production with *Ustilago trichophora* on minimal medium.** Depicted are **A** online data of OTR, **B** concentrations of phosphate and CDW, and **C** concentrations of sucrose, glucose, fructose, and malic acid over time. Cultivation was performed with *U. trichophora* in a 3 L benchtop fermenter using a minimal medium containing 0.18 g/L $KH_2PO_4$ and 75 g/L sucrose. For concentrations, mean values and standard deviations of technical triplicates are shown. Phosphate was only measured once using a Spectroquant cell cuvette kit ($PO_4$-P, Sigma Aldrich, Merck KgaA., Germany).

**Table S 1: Remaining substrate concentration, product titer, yield, and STY of batch processes in shake flasks.**

| Initial substrate concentration [g/L sucrose equivalents] | Remaining substrate concentration [g/L sucrose equivalents] | Product titer [g/L] | Yield [g/g] | STY [g/L/h] |
|---|---|---|---|---|
| 27 | n.d. | 8.9 ± 0.0 | 0.33 | 0.14 |
| 54 | n.d. | 17.0 ± 0.2 | 0.31 | 0.16 |
| 83 | n.d. | 20.4± 0.2 | 0.25 | 0.14 |
| 113 | 23.7 ± 0.7 | 22.4 ± 0.2 | 0.25 | 0.13 |

Cultivation conditions are given in Figure 2. Error of remaining substrate concentration and product titer is given as minima and maxima of biological duplicates.

**Table S 2: Succinic acid concentrations of shake flask experiments.**

| Experiment and sample specification | | Succinic acid [g/L] |
|---|---|---|
| Batch | | |
| | 27 g/L molasses | 2.4 ± 0.1 g/L |
| | 54 g/L molasses | 4.2 ± 0.0 g/L |
| | 83 g/L molasses | 4.1 ± 0.1 g/L |
| | 113 g/L molasses | 5.5 ± 0.0 g/L |
| | 135 g/L molasses | 0.4 ± 0.4 g/L |
| | 156 g/L molasses | 0.0 ± 0.0 g/L |
| Fed-Batch | | |
| | | 0.0 ± 0.0 g/L |
| Pulsed Batch | | |
| | Phase I | 4.1 g/L |
| | Phase II | 6.1 ± 0.0 g/L |

Cultivation conditions are given in Figure 2 and Figure 3. Concentrations are given as minima and maxima of biological duplicates.

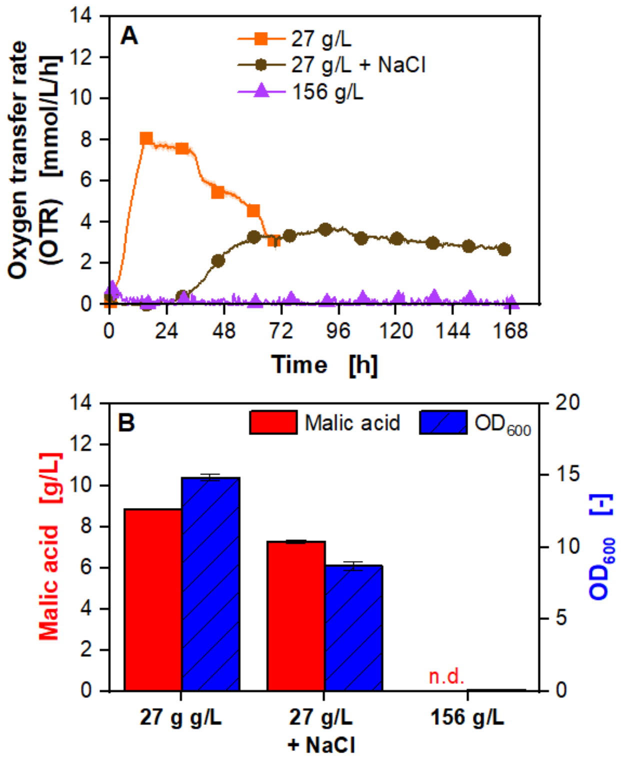


**Figure S 2: Influence of osmolality on malic acid production on molasses in shake flasks. A** Online data of OTR over time. For clarity, only every 30th data point is represented as a symbol. Lines are drawn through all measuring points. Shadows indicate the minimum and maximum values of biological duplicates. **B** Final malic acid concentration and $OD_{600}$. Samples were drawn after sugar was depleted, as indicated by the OTR (27 g/L) or after 168 h (27 g/L + NaCl, 156 g/L). Error bars indicate the minimum and maximum values of biological duplicates. Cultivation conditions are given in Figure 2. NaCl was added to one culture containing molasses equivalent to 27 g/L sucrose to achieve the same osmolality of 2.6 Osmol/kg as when using 156 g/L.

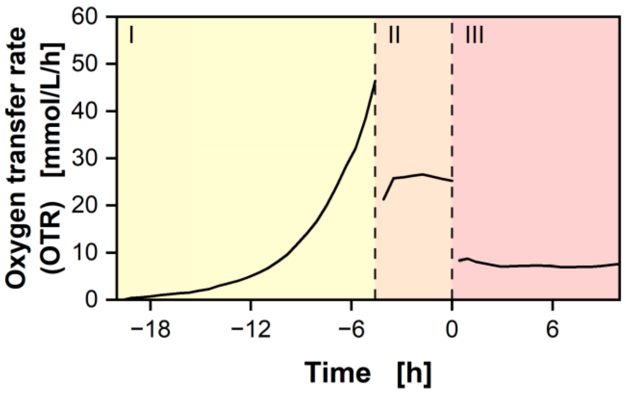


**Figure S 3: OTR of both precultures for the simulated fed-batch process in shake flasks.** I: First preculture for exponential growth of the organism. II: Second preculture to trigger malic acid production via secondary substrate limitation. III: First 10 h of fed-batch main culture. Complete data is shown in Figure 3. Cultivation conditions: RAMOS, I. Standard RAMOS flask, preculture medium, $OD_{600,\ Start}$ = 0.1 [-]. II. Standard RAMOS flask, adapted preculture medium, $OD_{600,\ Start}$ = 10 [-]. III. Fed-Batch flask, 0.3 M MES in initial medium, molasses equivalent to 230 g/L sucrose in reservoir, $OD_{600,\ Start}$ = 5 [-].

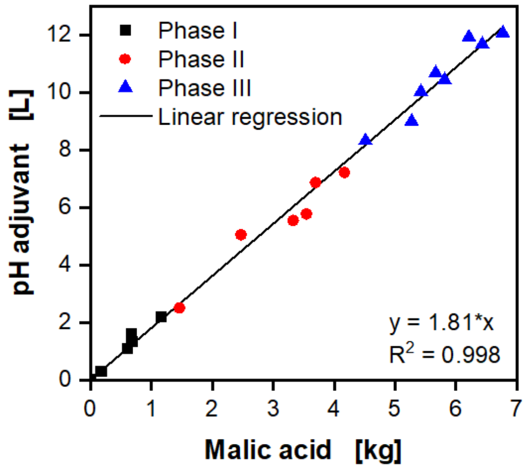


**Figure S 4: Linear correlation between produced malic acid and used pH adjuvant (10 M NaOH).** Data was taken from Figure 4.

**Process model in Aspen Plus**

The method "ELECNRTL" is applied to all unit operations in the process simulations, except for the crystallizers, which use "SOLIDS". UNIFAC is used to estimate binary interaction parameters. The following reactions are implemented in the model:

Equilibrium reactions:

$$H_2O \leftrightarrow H^+ + OH^-$$

$$H_2MA \leftrightarrow H^+ + HMA^-$$

$$HMA^- \leftrightarrow H^+ + HMA^{2-}$$

$$H_2SA \leftrightarrow H^+ + HSA^-$$

$$HSA^- \leftrightarrow H^+ + HSA^{2-}$$

Dissociation reactions:

$$NaOH \rightarrow Na^+ + OH^-$$

$$H_2SO_4 \leftrightarrow H^+ + HSO_4^-$$

$$HSO_4^- \leftrightarrow H^+ + SO_4^{2-}$$

Precipitation reactions:

$$Na_2SO_4 \leftrightarrow 2\ Na^+ + SO_4^{2-}$$

The protonated carboxylic acid species are added as user-defined molecular components via molecular structures, as they are not available in the component databank. Figure S 5 depicts the flowsheet in Aspen. Key parameters of each unit operation and inlet stream are listed in Table S 3.

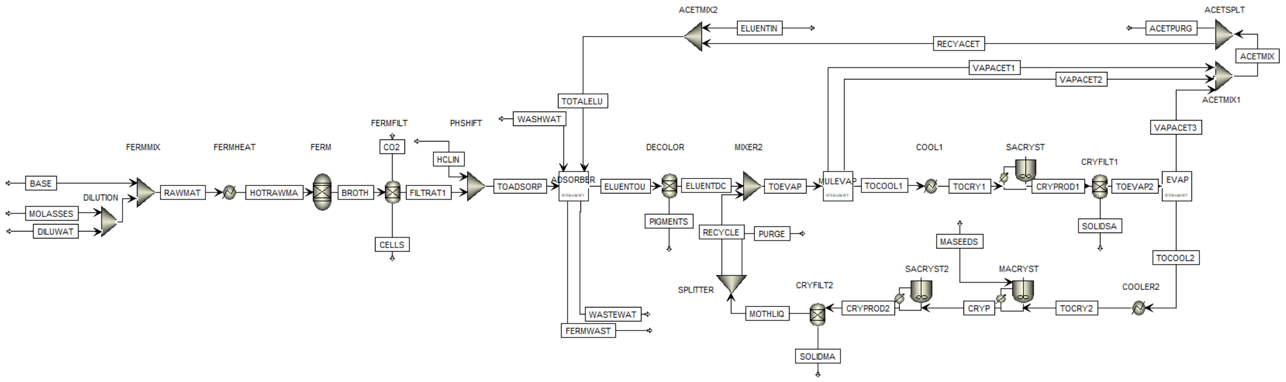


**Figure S 5: Aspen process flowsheet of the malic acid production process**

The molasses feed is simplified to a binary sucrose–water system (0.5 wt% sucrose). The base and water mass flows are parameterized by the final product titer and the fermentation pH. The fermenter is modelled as a RYield reactor, with the following de-lumping reaction at full sucrose conversion:

$$\text{Sucrose} \rightarrow 0.5\ \text{MA} + 0.4\ \text{CO2} + 0.1\ \text{SA}$$

In the subsequent separator, $CO_2$ and biomass are removed. Because biomass formation is not modeled, the stream CELLS is set to zero flow. The broth is adjusted to pH 2 and fed to the adsorption. Since cyclic adsorption cannot be modeled directly in Aspen Plus, the process is abstracted by a mixer (ADSORPT), which mixes the broth with acetone eluent, and a separation unit (DESORPT), which splits the streams according to experimental results. The adsorption model is shown in Figure S 6. Acetone is assumed to be the sole eluent. In DESORPT, all ionic acid species and inorganic salt ions are separated. Experimental data indicate adsorption yields of approximately 90% for malic acid and 98% for succinic acid. These yields are implemented by adjusting the split factors of the fully protonated acid species. Consequently, the outlet stream ELUENTOU contains acetone as well as fully protonated malic and succinic acids. Between each adsorption–desorption cycle, the column is washed, represented by the corresponding inlet and outlet water streams.

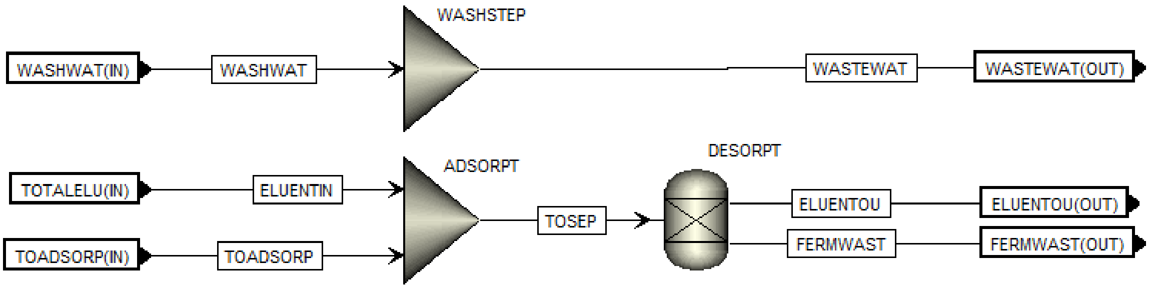


**Figure S 6: Aspen process flowsheet of the adsorption/desorption step**

After adsorption, the eluent is decolorized. However, since pigments are not modeled, the PIGMENTS stream is set to zero flow. The carboxylic acids are then concentrated by a two-step acetone evaporation with heat integration to minimize heat duty (Figure S 7). After the second evaporation, the malic acid concentration is 256.65 g/L, just below the solubility limit.

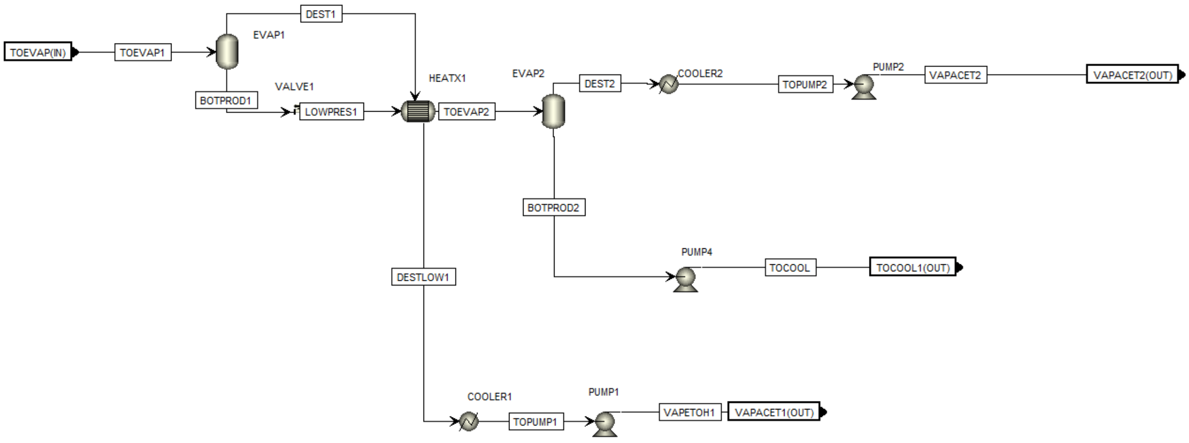


**Figure S 7: Aspen process flowsheet of the first evaporation step**

The outlet stream is cooled to 10°C and fed into the first crystallizer (SACRYST), where pure succinic acid crystallizes. The solid crystals are separated from the mother liquor by the filtration (CRYFILT1). The mother liquor is fed into the second evaporation step, which is shown in Figure S 8. The overhead

products of all three flash units contain pure acetone. These streams are cooled, collected (ACETMIX1), and recycled to adsorption, with a 0.1% purge (ACETPURG).

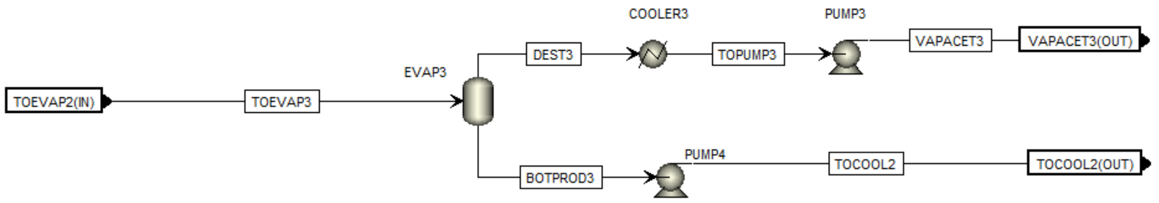


**Figure S 8: Aspen process flowsheet of the second evaporation step**

The concentrated malic acid stream TOCOOL1 is cooled to 10°C and fed into the second crystallizer MACRYST, where malic acid crystallizes. Because Aspen Plus cannot crystallize two products in a single unit, a third crystallizer (SACRYST2) follows, in which succinic acid crystallizes. The combined solid product is removed from the mother liquor by filtration (CRYFILT2). Finally, 90% of the mother liquor is recycled.

**Table S 3: Key parameters of unit operations and inlet streams**

| Unit Operation/Stream | Parameter | Value |
|---|---|---|
| **Streams** | | |
| INPUT BASE | Mass flow | 4.03 $kg/kg_{Product}$ |
| | Mass fraction NAOH | 0.17 |
| INPUT MOLASSES | Mass flow | 3.80 $kg/kg_{Product}$ |
| | Mass fraction SUCROSE | 0.5 |
| INPUT DILUWAT | Mass flow | 3.43 $kg/kg_{Product}$ |
| | Mass fraction WATER | 1 |
| INPUT H2SO4 | Mass flow | 0.87 $kg/kg_{Product}$ |
| | Mass fraction H2SO4 | 0.95 |
| INPUT ELUENTIN | Mass flow | 0.02 $kg/kg_{Product}$ |
| | Mass fraction ACET | 1 |

| Unit Operation/Stream | Parameter | Value |
| --- | --- | --- |
| INPUT WASHWAT | Mass flow | 8.63 $kg/kg_{Product}$ |
| | Mass fraction WATER | 1 |
| INPUT MASEEDS | Mass flow | 0.02 $kg/kg_{Product}$ |
| | Mass fraction SOLID MA | 1 |
| **Heat exchanger** | | |
| FERMHEAT | Outlet temperature | 30°C |
| | Outlet pressure | 1 bar |
| MULEVP.COOLER1, MULEVP.COOLER2, EVAP.COOLER3 | Outlet temperature | 35°C |
| | Outlet pressure | 1 bar |
| COOL1, COOLER2 | Outlet temperature | 10°C |
| | Outlet pressure | 1 bar |
| HEATX | Cold stream outlet temperature | 41°C |
| | Minimum temperature approach | 10°C |
| **Pressure changers** | | |
| MULEVAP.PUMP1, MULEVAP.PUMP2, MULEVAP.PUMP3, EVAP.PUMP3, EVAP.PUMP4 | Outlet pressure | 1 bar |
| MULEVAP.VALVE1 | Outlet pressure | 0.45 bar |
| **Splitter** | | |
| ACETSPLIT | Split fraction to purge | 0.001 |
| SPLITTER | Split fraction to purge | 0.01 |
| **Separators** | | |
| FERMFILT: Split fraction to FILTRATE1 | $CO_2$ | 0 |
| | Solids | 0 |
| | Other components | 1 |
| DECOLOR: Split fraction to ELUENTDC | All components | 1 |

| Unit Operation/Stream | Parameter | Value |
| --- | --- | --- |
| DESORPT: Split fraction to ELUENTOU | H2MA | 0.9 |
| | H2SA | 1 |
| | ACET | 1 |
| | Other components | 0 |
| CRYFILT1, CRYFILT2: Split fraction to SOLIDSA/SOLIDMA | SOLDIS | 1 |
| | Other components | 0 |
| **Flash** | | |
| MULEVAP.EVAP1 | Pressure | 1 bar |
| | Temperature | 57.25°C |
| MULEVAP.EVAP2 | Pressure | 45 bar |
| | Temperature | 37°C |
| EVAP.EVAP3 | Pressure | 0.18 bar |
| | Temperature | 35°C |
| **Reactor** | | |
| FERM | De-lumping coefficient MA | 0.5 |
| | De-lumping coefficient SA | 0.1 |
| | De-lumping coefficient $CO_2$ | 0.4 |
| **Crystallizer** | | |
| MACRYST, SACRYST, SACRYST2 | Pressure | 1 bar |
| | Temperature | 10°C |

**Table S 4: Stream and Utility prices**

| Stream/Utility | Composition | Price | Source |
|---|---|---|---|
| Molasses | 50 wt% | 130 EUR/tonne | Personal communication |
| Aceton | 100 wt% | 640 EUR/tonne | [1][1] |
| Process water | 100 wt% | 1.4 EUR/tonne | [2] |
| NaOH | 17 wt% | 50 EUR/tonne | [3][1] |
| $H_2SO_4$ | 95 wt% | 100 EUR/tonne | [4][1] |
| Malic acid | 100 wt% | 1174 EUR/tonne | [5][1] |
| Succinic acid | 100 wt% | 1204 EUR/tonne | [6][1] |
| $CO_2$ (Process and utility) | | 82 EUR/tonne | [7][1] |
| Waste | | 2.9 EUR/tonne | [2] |
| Low pressure steam | | 27 EUR/tonne | [2] |
| Cooling Water | | 0.05 EUR/kWh | [2] |
| Electricity | | 0.2 EUR/kWh | [8][1] |

[1] Exchange rate: 0.86 EUR/USD [9]

## References


1. Chemanalyst. Acetone Price Trend and Forecast. 2025. https://www.chemanalyst.com/Pricing-data/acetone-12. Accessed 3 Dec 2025.
2. Saur K, Kiefel R, Niehoff P, Hofstede J, Ernst P, Brockkötter J, et al. Holistic approach to process design and scale-up for itaconic acid production on complex substrates. MDPI. 2023;10.
3. Chemanalyst. Caustic Soda Price Trend and Forecast. 2025. https://www.chemanalyst.com/Pricing-data/caustic-soda-3. Accessed 3 Dec 2025.
4. Chemanalyst. Sulphuric Acid Price Trend and Forecast. 2025. https://www.chemanalyst.com/Pricing-data/sulphuric-acid-70. Accessed 3 Dec 2025.
5. Chemanalyst. Malic Acid Price Trend and Forecast. 2025. https://www.chemanalyst.com/Pricing-data/malic-acid-1281. Accessed 3 Dec 2025.

6. Chemanalyst. Succinic Acid Price Trend and Forecast. 2025. https://www.chemanalyst.com/Pricing-data/succinic-acid-1270. Accessed 3 Dec 2025.
7. Trading Economics. EU Carbon Permits. 2025. https://tradingeconomics.com/commodity/carbon. Accessed 3 Dec 2025.
8. Statista. Industriestrompreise in Deutschland in den Jahren 2000 bis 2024 (in Euro-Cent pro Kilowattstunde). 2025. https://de.statista.com/statistik/daten/studie/155964/umfrage/entwicklung-der-industriestrompreise-in-deutschland-seit-1995/. Accessed 3 Dec 2025.
9. Statista. Euro (EUR) to U.S. dollar (USD) exchange rate from March 13, 2018 to November 14, 2025. https://www.statista.com/statistics/412794/euro-to-u-s-dollar-annual-average-exchange-rate/. Accessed 3 Dec 2025.
